# The Novel Antitumor Compound HCA Promotes Glioma Cell Death by Inducing Endoplasmic Reticulum Stress and Autophagy

**DOI:** 10.3390/cancers13174290

**Published:** 2021-08-26

**Authors:** Roberto Beteta-Göbel, Javier Fernández-Díaz, Laura Arbona-González, Raquel Rodríguez-Lorca, Manuel Torres, Xavier Busquets, Paula Fernández-García, Pablo V. Escribá, Victoria Lladó

**Affiliations:** 1 Laboratory of Molecular Cell Biomedicine, Department of Biology, University of the Balearic Islands, 07122 Palma de Mallorca, Spain; roberto.beteta@uib.es (R.B.-G.); j.fernandez@uib.es (J.F.-D.); laura.arbona@uib.es (L.A.-G.); raquel.rodriguez@uib.es (R.R.-L.); manuel.torres@uib.es (M.T.); xavier.busquets@uib.es (X.B.); pablo.escriba@uib.es (P.V.E.); 2 Department of R&D, Laminar Pharmaceuticals, Isaac Newton, 07121 Palma de Mallorca, Spain; paula.fernandez@uib.es

**Keywords:** HCA, membrane lipid therapy, glioma, ER stress, autophagy

## Abstract

**Simple Summary:**

Melitherapy is an innovative therapeutic approach to treat different diseases, including cancer, and it is based on the regulation of cell membrane composition and structure, which modulates relevant signal pathways. In this context, 2-hydroxycervonic acid (HCA) was designed for patients with cancer or other pathologies who have received ineffective and safe treatment. Here, we have tested the effects of HCA on glioblastoma cells and xenograft tumors (mice). HCA appeared to enhance endoplasmic reticulum stress/unfolded protein response signaling, which consequently induced autophagic cell death of the glioblastoma tumor cells. In light of the data obtained, it would clearly be worthwhile to undertake more clinically orientated studies to fully assess the potential of HCA to combat glioblastoma in patients.

**Abstract:**

Glioblastoma (GBM) is the most common and aggressive type of primary brain tumor in adults, and the median survival of patients with GBM is 14.5 months. Melitherapy is an innovative therapeutic approach to treat different diseases, including cancer, and it is based on the regulation of cell membrane composition and structure, which modulates relevant signal pathways. Here, we have tested the effects of 2-hydroxycervonic acid (HCA) on GBM cells and xenograft tumors. HCA was taken up by cells and it compromised the survival of several human GBM cell lines in vitro, as well as the in vivo growth of xenograft tumors (mice) derived from these cells. HCA appeared to enhance ER stress/UPR signaling, which consequently induced autophagic cell death of the GBM tumor cells. This negative effect of HCA on GBM cells may be mediated by the JNK/c-Jun/CHOP/BiP axis, and it also seems to be provoked by the cellular metabolite of HCA, C21:5n-3 (heneicosapentaenoic acid). These results demonstrate the efficacy of the melitherapeutic treatment used and the potential of using C21:5n-3 as an efficacy biomarker for this treatment. Given the safety profile in animal models, the data presented here provide evidence that HCA warrants further clinical study as a potential therapy for GBM, currently an important unmet medical need.

## 1. Introduction

Glioblastoma (GBM) is the most common and aggressive primary brain tumor in adults [1,2]. Based on its histopathological characteristics, including necrosis and endothelial proliferation, the World Health Organization (WHO) considers GBM a grade IV cancer, the highest grade of brain tumors [3]. The standard treatment for GBM includes surgery, radiotherapy, and alkylating chemotherapy with Temozolomide (TMZ: [4]). Nevertheless, at least 50% of patients fail to respond to TMZ due to the over-expression by down-methylated promotor of O^6^-methylguanyl DNA methyltransferase (MGMT) and/or deficiencies in the DNA repair pathway in GBM cells [5]. Moreover, GBM has a remarkably poor prognosis, showing a 5-year survival rate of 4–5%, and in clinical trials, a survival rate at 2 years of only 26–33% [2]. Hence, there is a clearly a clinical requirement to develop new therapies that better combat GBM.

Melitherapy (Membrane-Lipid Therapy) is an innovative therapeutic approach to treat different diseases, including cancer, and it is based on modifying the composition and structure of cell membranes [6]. The first rationally designed molecule to be used in this field, 2OHOA (2-hydroxyoleic acid, C18:1 n-9), is a bioactive lipid proven to be efficacious against and safe to administer to patients with glioma and other advanced solid tumors in a phase I/IIa clinical trial [7] (ClinicalTrials.gov identifier #NCT01792310). Another molecule that can be utilized in this pioneering approach is HCA (2-hydroxycervonic acid, 2-hydroxydocosahexaenoic acid, C22:6 n-3), a novel hydroxyl-derivative of DHA (docosahexaenoic acid, 22:6 n-3) that bears a hydroxyl group on the α-carbon [8]. DHA is the most abundant omega-3 polyunsaturated fatty acid (PUFA) in the brain and it is involved in the functioning of the central nervous system (CNS) [9]. As is well-known, lipid metabolism is deregulated in cancer cells. Specifically, a reduction of DHA levels has been observed in glioma tissue compared to normal brain tissue [10]. Significantly, omega-3 PUFAs such as DHA and EPA (eicosapentaenoic acid, C20:5 n-3) also have proven anti-cancer properties in vitro and in vivo, although the mechanisms underlying these benefits are not clear [11]. For instance, the antitumor effect of DHA (250 mg/kg/day) or in combination with cisplatin has been demonstrated in vivo in the Ehrlich Ascites Carcinoma solid tumor mice model [12]. Due to these anti-cancer properties, clinical trials have been developed to assess the potential of using fish oil or n-3 PUFAs to prevent and treat cancer [13]. In fact, there are clinical trials investigating the benefit of DHA supplementation (4.4 g/day of DHA, orally) in combination with neoadjuvant chemotherapy in breast cancer patients [14], but its benefits as an antitumor drug are still unclear. As such, HCA may potentially be suitable to treat neurodegenerative diseases in the CNS and/or cancer, due to its activity as an omega-3 PUFA, but with a different metabolization pathway, compared to DHA [8,15,16,17].

In many diseases, misfolded proteins accumulate in the endoplasmic reticulum (ER) of different tissues, such as cancer. Upon the accumulation of these aberrant proteins, cells will activate the unfolded protein response (UPR) to restore proteostasis, involving the activation of three ER transmembrane proteins: protein kinase R-like ER kinase (PERK), inositol-requiring enzyme 1 (IRE1α), and activating transcription factor 6α (ATF6) [18]. Under basal conditions, these proteins associate with the ER-resident glucose-regulated protein 78 (GRP78 or BiP), and their dissociation from these complexes in conditions of ER stress aims to restore ER proteostasis [19]. However, if this ER proteostasis cannot be restored, cell death will be triggered via processes such as apoptosis or autophagy [20].

Autophagy is a means of degrading and recycling dysfunctional cell components, whereby subcellular membranes are reorganized to sequester portions of the cytoplasm and organelles into autophagosomes, structures that will be transported to the lysosome to degrade the elements captured [21]. Autophagy is a highly conserved process, tightly controlled by autophagy-related genes (ATGs) such as LC3B [22] and by the autophagosome cargo protein (p62/sequestosome 1), a protein that binds to degradation targets and facilitates selective autophagy [23]. Dysfunctional autophagy contributes to many diseases, and in cancer, autophagy can be neutral, tumor-suppressive, or tumor-promoting in different contexts [24]. Moreover, excess autophagy can induce a form of cell death known as autophagic cell death [25], and indeed, 2OHOA induces ER stress/UPR and autophagy in human gliomas [26].

As a result, we have studied the effects of HCA on GBM, showing that this hydroxyl-derivative of DHA regulates cell signaling and triggers GBM cell death through UPR and autophagy. In essence, treatment with HCA appears to represent an interesting novel anti-tumoral candidate for the treatment of GBM.

## 2. Materials and Methods

### 2.1. Cell Culture

U-118 MG (ATCC^®^ HTB-15™) cells were obtained from the American Type Culture Collection (Maassas, VA, USA), and other GBM cancer cell lines were obtained from Apointech SI (Salamanca, Spain) and cultured in RPMI 1640 medium (Sigma-Aldrich, St Louis, MO, USA), supplemented with 10% Fetal Bovine Serum (FBS, Sigma-Aldrich). HCA and DHA were obtained from Medalchemy (Alicante, Spain). The cells were incubated at 37 °C in an atmosphere of 5% CO_2_. Cell viability was determined using the Trypan blue staining method.

### 2.2. Animals, Tumor Xenografts, and Treatments

Male and female NUDE (Swiss) Crl:NU (Ico)-Foxn1^nu^ mice (8-weeks-old, 25–35 g; Charles River Laboratories, Paris, France) were maintained on a 12 h dark/light cycle and in a thermostat cabinet (28 °C: EHRET, Labor-U-Pharmatechnik, Tulln an der Donau, Austria), with a sterile air flow at a relative humidity of 40–60%. Autoclaved food and water were supplied ab libitum. Inoculation of U-118 MG (ATCC^®^ HTB-15™) xenograft tumors was performed as described previously in [27]. Briefly, 7.5 × 10^6^ cells were subcutaneously inoculated into both sides of the animals’ dorsal flank, and tumors became visible after two weeks. Tumors were measured weekly with a caliper, and tumor volumes (v) were calculated as v = W^2^ × L/2, where W is the tumor width and L is its length. Mice were orally administered the following compounds every day for 42 days: vehicle alone (5% etanol), HCA (200 mg/kg), or TMZ (80 mg/kg). All the protocols employed were approved by the Bioethical Committee at the University of the Balearic Islands, and they are in accordance with national and international guidelines on animal welfare.

### 2.3. Cell Proliferation Assays

Metabolically active cells were analyzed using the cell proliferation kit II (Roche, Basel, Switzerland), according to the manufacturer’s instructions, and this procedure was performed as described previously in [27].

### 2.4. Cell Transfection, Gene Expression Silencing, and Commercial Inhibitors

The ptfLC3 plasmid used here was a generous gift from Tamotsu Yoshimori (Addgene plasmid #21074; http://n2t.net/addgene:21074; accessed on: 30 January 2018; RRID: Addgene_21074, Watertown, MA, USA) [28]. Lipofectamine 2000 (ThermoFisher, Barcelona, Spain) was used to achieve transient transfection following the manufacturer’s instructions. The silencing of CHOP gene expression was performed by transient transfection of commercial siRNAs from Dharmacon (#J-004819-06, #J-004819-07, and #J-004819-08), which were introduced into the cells using RNAiMax (Life Technologies, Barcelona, Spain) during 24 h incubations. The DDIT3 sequence of CHOP was used to design the siRNA (accession number NM_004083 NCBI) and a negative siRNA control (siNeg) from Life Technologies was used as a control (unknown mixed sequences). The commercial inhibitors employed were: 3-methyladenine (3-MA, 5 mM; Merck KGaA, Darmstadt, Germany), Bafilomycin A1 (BafA1, 10 nM, Sigma), LY294002 (25 µM, Biogen Cientifica, Madrid, Spain), Oxythiamine chloride hydrochloride (OT, 1, 10 mM, or 100, 200, or 300 mg/kg mice; Santa Cruz Biotechnology, Heidelberg, Germany), and SP600125 (25 µM, Santa Cruz Biotechnology). The cells were seeded in culture plates and pre-treated with these commercial inhibitors for 1.5 h prior to their exposure to HCA for different times.

### 2.5. Cell Lysis, Electrophoresis, and Immunoblotting and Protein Quantification

Cells were washed with PBS, and protein extraction buffer (10 mM Tris-HCl, 2 mM EDTA, 1% SDS, 5 mM Protease Inhibitor Complete (Roche), and 1 mM Sodium Orthovanadate) was added to each plate. The protein in the extract was quantified and immunoblotting was performed as described previously [29], with minor modifications. The membranes were probed overnight with primary antibodies diluted in fresh blocking solution, against: ATF-6, BiP, CHOP, p-eIF2α (Ser51), Ireα, p-c-Jun (Ser63), LC3B, p-SAPK/JNK (Thr183/Tyr185: 1:1000, Cell Signaling, Leiden, The Netherlands), ATF-4 (1:2000, ProteinTech, Manchester, UK), eIF2α, PARP (1:1000, Santa Cruz Biotechnology), c-Jun (1:1000, BD-Biosciences, Madrid, Spain), and SQSTM1/p62 (1:5000, Thermo-Scientific/Pierce, Barcelona, Spain). Membranes were incubated for 1 h at room temperature with secondaries antibodies (anti-mouse or anti-rabbit IgG, Li-cor, Biosciences, Lincoln, NE, USA) diluted in blocking solution (1:5000). Finally, the protein detected was quantified by integrated photo-densitometry after near-infrared scanning at 700 nm (Odissey, Li-cor Biosciences), using α-Tubulin (1:10,000, Sigma) as a loading control. The bars in the figures represent the average quantification from several experiments, each one with various replicates. The values in each lane were normalized to the tubulin content, and normalized values in the treated cells were considered relative to those of the untreated control and non-transfected cells.

### 2.6. Immunofluorescence and Confocal Microscopy

Cells were seeded onto 25 mm circular coverslips in 24-well plates at a density of ca. 25 × 10^3^ cells/well in 1 mL of RPMI 1640 medium supplemented with 10% FBS. The cells were exposed to HCA for 48 h. Cell fixation and immunostaining were performed as described previously [26]. In some cases, F-actin was labeled with Alexa Fluor 488 phalloidin.

### 2.7. Cathepsin Assay

Cathepsin B activity was analyzed using the Kit InnoZymeTM Cathepsin B Activity Assay (Merck KGaA), following the manufacturer’s instructions.

### 2.8. Fatty Acid Analysis by Gas Chromatography (GC)

Glioma cells were seeded in 10 cm diameter culture plates at a density of 1 × 10^6^ cells/plate and maintained in the presence or absence of the compounds for different periods. Lipids were extracted directly from the cell lysates or tumors, as described previously in [27].

### 2.9. Data Analysis

The data were expressed as the mean ± SEM or the % relative to the untreated controls from independent experiments, as indicated in each figure. To determine the significance of the differences, a Student’s *t*-test (two groups) or a one-way ANOVA (comparison between several groups) were used (GraphPad Prism 6.1). The level of significance was set at 95% confidence (*p* < 0.05). 

## 3. Results

### 3.1. Efficacy of HCA against GBM

**Figure 1:**
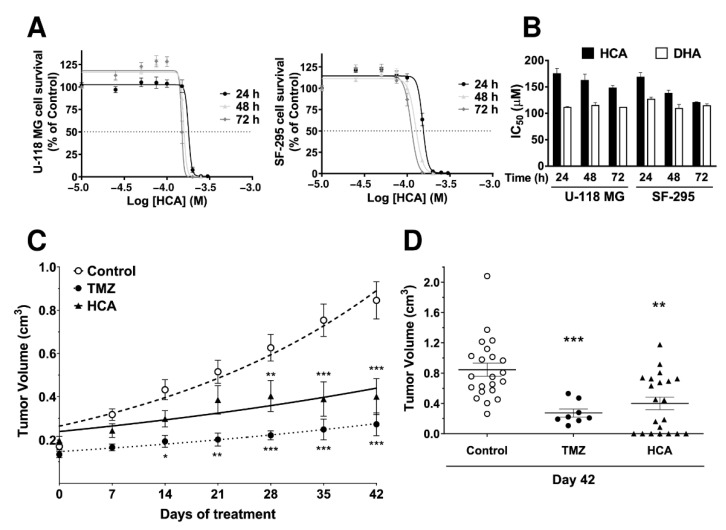
Efficacy of HCA against human glioma. (**A**) Time- and concentration-dependent inhibition of human glioma (U-118 MG and SF-295) cell growth by HCA. (**B**) IC_50_ values for HCA and DHA against glioma cell proliferation after a 24, 48, and 72 h treatment (mean ± SEM from 3 independent experiments performed in quadruplet). (**C**) Effects of the vehicle alone (control), HCA (200 mg/kg, p.o., daily), or TMZ (80 mg//kg, p.o., daily) against U-118 MG-derived tumor growth in mice during a 42-day treatment. (**D**) Tumor volumes after a 42-day treatment (mean ± SEM: Control, 22 tumors (*n* = 13, 3 males and 10 females). HCA, 21 tumors (*n* = 12, 6 males and 6 females); TMZ, 8 tumors (*n* = 4, 1 male and 3 females). Student’s t-test: *** *p* < 0.001, ** *p* < 0.01, * *p* < 0.05 with respect to the controls. HCA: 2-hydroxycervonic acid; DHA: docosahexaenoic acid; TMZ: temozolomide.

The efficacy of HCA against glioma was tested using different human GBM cell lines: U-118 MG, SF-295, SF-268, SNB-19, SNB-75, and U-251. All these cell lines showed standard growth dynamics in the absence of HCA, while exposure to 100–200 µM of HCA induced a time- and concentration-dependent inhibition of cell growth, and GBM cell death (Figure 1A,B and Supplementary Table S1). HCA and DHA provoked a reduction in GBM cell number at all the time points studied (Figure 1B), and the IC_50_ values for HCA ranged from 175 ± 10.1 µM in U-118 MG after 24 h to 109.3 ± 7.5 µM after 72 h for SF-295 cells, relatively similar to 126.7 ± 4.1 µM after 24 h in SF-295 cells for DHA to 115 ± 5.5 µM after 72 h in U-118 MG cells. However, it was notable that while the IC_50_ values of DHA remained relatively constant over the 72 h of the assay with both cell types, the IC_50_ of HCA diminished over time in the case of both cell lines. A similar temporal decline was also observed with the other cell lines tested (Supplementary Table S1).

The efficacy of HCA against GBM was tested in vivo using a U-118 MG cell xenograft model of GBM [30,31]. HCA (200 mg/kg, p.o., daily) induced a marked and significant reduction in the volume of U-118 MG tumors, independently of the mice sex, with some tumors even disappearing after this treatment (Figure 1C,D). However, it appeared that TMZ treatment (80 mg/kg, p.o., daily), the standard therapy for GMB, caused an even greater reduction in tumor volume than HCA by day 42 (control, 0.84 ± 0.08 cm^3^; HCA, 0.40 ± 0.08 cm^3^; TMZ, 0.27 ± 0.05 cm^3^, Figure 1D), and although the use of TMZ increases the median survival in patients with glioma by 10 weeks [4], at least 50% of patients fail to respond to TMZ [5]. Significantly, and unlike HCA, TMZ was not capable of producing the complete disappearance of the tumors in any instance (Figure 1D). During these experiments, no changes in the weight or mortality of the mice were detected between the groups of mice that received TMZ or HCA, although both drugs produced a minor loss in weight relative to the control mice (Supplementary Figure S1). Similarly, no side effects were observed in association with HCA, indicating that its oral administration was well-tolerated. Indeed, toxicity studies performed previously with HCA on zebrafish, *Drosophila melanogaster,* and mice highlighted its absence of toxicity [8,15,32].

### 3.2. HCA Activates ER Stress/UPR Signaling Pathways in GBM Cells

**Figure 2:**
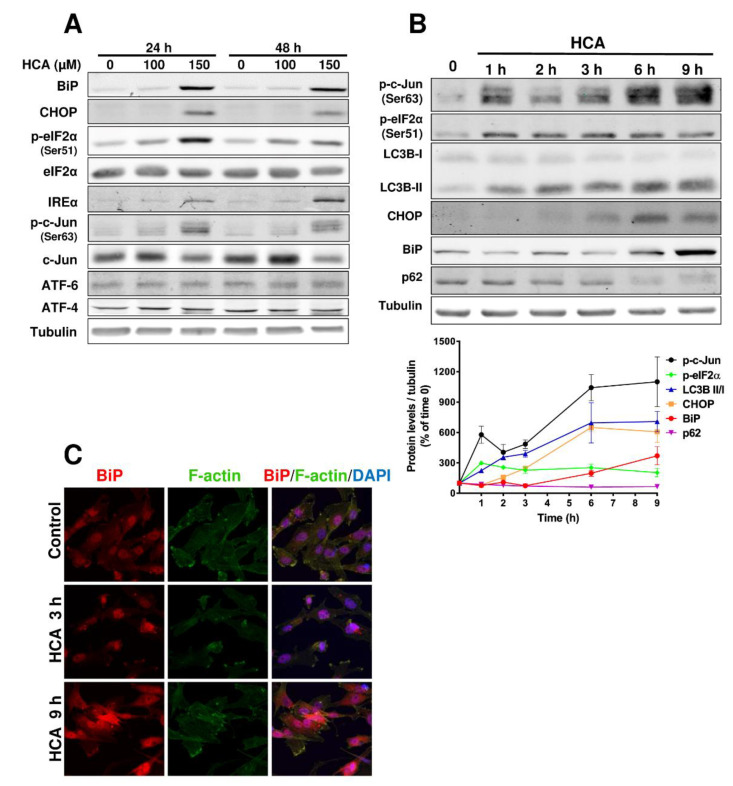
HCA activates ER stress/UPR signaling pathways in U-118 MG GBM cells. (**A**) Representative immunoblots of the effect of HCA (100 or 150 µM for 24 or 48 h) on eIF2α and c-Jun phosphorylation, and on the BiP, CHOP, ATF6, ATF4, and IREα proteins, and the amounts of tubulin in U-118 MG cells as a loading control. (**B**) U-118 MG cells were treated with 200 µM of HCA for 1, 2, 3, 6, and 9 h, and protein levels or phosphorylation were determined in immunoblots (left) and quantified (mean ± SEM of three independent experiments, right). (**C**) BiP immunofluorescence (red) in U-118 MG cells treated with 150 µm of HCA for 3 or 9 h. F-actin was labeled with Alexa Fluor 488 phalloidin and the nuclei with DAPI. Representative micrographs (single confocal planes) are shown: scale bar, 5 μm. HCA: 2-hydroxycervonic acid; ATF: Activating Transcription Factor; BiP: a.k.a. GRP78, glucose-regulated protein 78; CHOP: a.k.a. DDIT3, DNA Damage Inducible Transcript 3; c-Jun: Jun Proto-Oncogene, AP-1 Transcription Factor Subunit; eIF2a: Eukaryotic Translation Initiation Factor 2A; IREα: inositol-requiring enzyme 1; LC3B: a.k.a. ATG8F, Microtubule Associated Protein 1 Light Chain 3 Beta; p62: a.k.a. SQSTM1, Sequestosome 1.

We questioned whether the inhibition of U-118 MG and SF-295 cell growth by HCA might be mediated by ER stress/UPR signaling, examining the expression of key molecules in the three main signal transduction cascades activated by ER stress/UPR. When U-118 MG and SF-295 cells were treated with increasing concentrations of HCA (100 and 150 µM) for 24 and 48 h, induction of the BiP and CHOP (DDIT3, DNA Damage-Inducible Transcript 3) protein levels were observed in Western blots (Figure 2A, Supplementary Figure S2A). Furthermore, exposure to HCA increased the phosphorylation of eIF2α (Ser51) and c-Jun (Ser63), and upregulated IREα, ATF6, and ATF4 in SF-295 cells (Supplementary Figure S2A).

To establish the temporal sequence in which the different signaling pathways are activated in cells upon treatment with HCA, cells were treated with HCA (200 µM) and collected at different times thereafter (from 1 to 9 h after exposure to HCA: Figure 2B). A significant increase in phosphorylated c-Jun (Ser63) and eIF2α (Ser51) proteins was observed one hour after exposure to HCA, as were the LC3B levels. CHOP induction was observed 2 h after exposure to HCA, and moreover, the increases in CHOP, phospho-c-Jun, and LC3B reached their maximums in U-118 MG cells after 9 h (607 ± 103%, 1100 ± 244%, 707 ± 100%, compared to baseline, respectively) or after 12 h in SF-295 cells (946 ± 415%, 735 ± 120%, 2425 ± 385%, compared to baseline, respectively: Figure 2B, Supplementary Figure S2B). In contrast, eIF2α phosphorylation remained constant after 1 h in the presence of HCA (U-118 MG, between 297 ± 11% and 203 ± 43%; SF-295, between 268 ± 110% and 214 ± 77%). For the p62 protein, two opposing responses were observed in the two cell lines, with a loss of this protein in U-118 MG cells, while it augmented in SF-295 cells over time. Finally, the amount of the BiP chaperone detected in immunoblots increased from 6 h after incubation with HCA, and its location in the different cell compartments (nucleus, cytosol, and membrane) was altered in the presence of this drug relative to untreated cells, as evident in confocal microscopy images. When the cells were treated with HCA for 3 h, BiP accumulated in the nucleus, and there was a considerable increase of BiP in the whole cell, indicating that ER stress was enhanced after 9 h of treatment relative to the cells not exposed to HCA (Figure 2C).

### 3.3. HCA Induces Autophagy in GBM Cells

**Figure 3:**
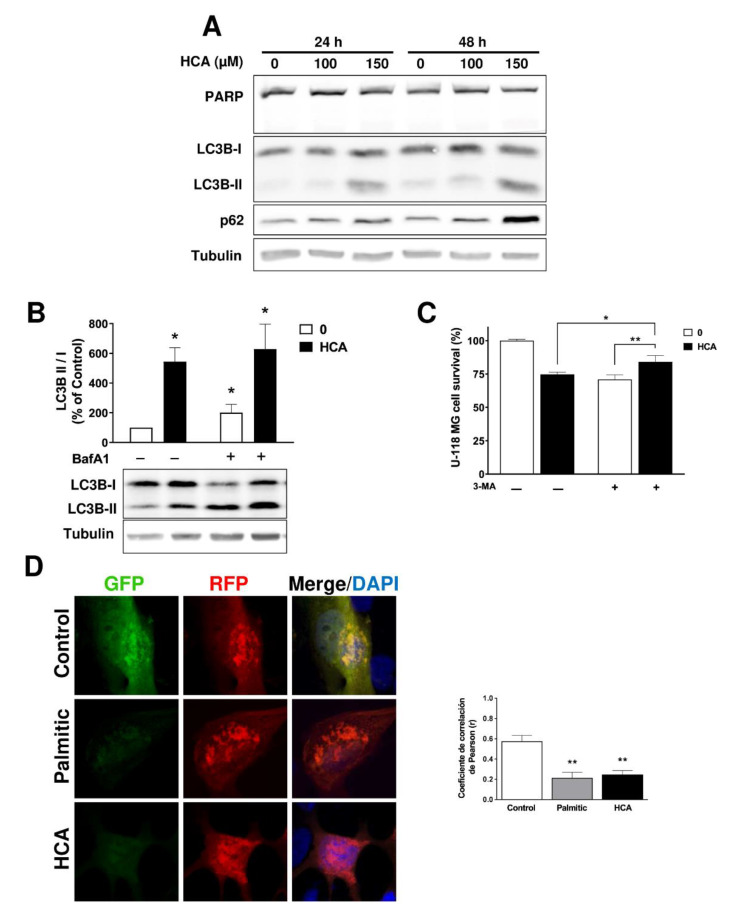
HCA induces autophagy in U-118 MG GBM cells. (**A**) Representative immunoblots of the effect of HCA (100 or 150 µM for 24 or 48 h) on the PARP, LC3B, and p62 proteins in U-118 MG cells, using tubulin as a loading control. (**B**) Effect of bafilomycin A1 (BafA1) on the LC3B-II levels in HCA-treated cells. (**C**) Effect of 3-Methyladenine (3-MA) on the survival of HCA-treated U-118 MG cells. The cells were pretreated with BafA1 (10 nM) or 3-MA for 1.5 h before exposure to HCA (150 µM) for 48 h (bars correspond to the mean ± SEM values of 3 independent experiments). (**D**) Monitoring autophagy flux. The cells were transfected with the ptfLC3 plasmid expressing LC3 tagged with GFP and RFP, and then treated with palmitic acid (150 µM, positive control of autophagy) or HCA (150 µM) for 48 h. Nuclei were labeled with DAPI. Representative micrographs (single confocal planes) are shown: scale bar, 5 μm (left). Co-localization analysis of autophagosomes (green) and autolysosomes (red) using a Pearson correlation coefficient (r: bars correspond to the mean ± SEM values of 6 cells, right). Student’s t-test: ** *p* < 0.01, * *p* < 0.05 with respect to the control. HCA: 2-hydroxycervonic acid; BafA1: bafilomycin A1; 3-MA: 3-Methyladenine; GFP: Green Fluorescent Protein; RFP: Red Fluorescent Protein; LC3B: a.k.a. ATG8F, Microtubule Associated Protein 1 Light Chain 3 Beta; p62: a.k.a. SQSTM1, Sequestosome 1; PARP: Poly(ADP-Ribose) Polymerase; +: presence of the drug indicated in the figure; –: absence of the drug indicated in the figure.

In order to determine the mechanisms activated by HCA that provoke cell death and reduce the viability of GBM cells, markers of different cell death pathways were explored. The U-118 MG and SF-295 cells were treated with HCA (100 and 150 µM) for 24 or 48 h, and the accumulation of different cell markers was analyzed in Western blots. Proteolytic cleavage of PARP into fragments by caspases is an early indicator of apoptosis [33], yet there was no evidence that exposing cells to HCA produced any loss of PARP, indicating that apoptosis was not a major influence on U-118 MG cell viability (Figure 3A), or on that of SF-295 cells (Supplementary Figure S3A). Upon induction of autophagy, LC3B-I is converted to LC3B-II by an ubiquitin-like conjugation system that attaches a phosphatidylethanolamine moiety to LC3B-II [34]. When the cells of either cell line were exposed to HCA, a marked increase in LC3B-II was evident (Figure 3A, Supplementary Figure S3A). Moreover, SQSTM1/p62 is a selective autophagy receptor that sequesters ubiquitinated proteins into autophagic vacuoles by interacting with LC3, and it is a substrate for autophagic degradation [35]. HCA provoked an increase in p62 levels in both cell lines when it was analyzed after 24 or 48 h of treatment (Figure 3A, Supplementary Figure S3A), providing further evidence that these cells underwent autophagy when exposed to this drug. Furthermore, confocal microscopy revealed the cytoplasmic aggregation of LC3 upon exposure to HCA but not DHA, suggesting that these molecules have different mechanisms of action (Supplementary Figure S3B).

The increase in LC3B-II might be associated with either enhanced autophagosome synthesis or reduced autophagosome turnover. To distinguish between these two possibilities, cells were treated with bafilomycin A1 (BafA1) to inhibit vacuolar H1-ATPase in the late phase of autophagy, thereby impeding lysosome acidification and increasing the amount of LC3B-II/I in cells. In the presence of BafA1, there was an increase in LC3B-II in both control and HCA-treated cells (Figure 3B), indicative of efficient autophagic signal propagation in both situations. By blocking autophagosome formation via the inhibition of class III PI3K, 3-Methyladenine (3-MA) inhibits autophagy [36], and as such, 3-MA reversed the anti-proliferative effect of HCA on U-118 MG cells. Indeed, cell survival increased from 74.8 ± 1.6% in the presence of HCA alone to 84.1 ± 4.7% survival in the presence of both 3-MA and HCA (Figure 3C). Significantly, exposure of the cells to 3-MA alone affected cell viability to a similar extent as HCA.

Autophagic flux was monitored using the ptfLC3 plasmid that expresses LC3 tagged with GFP and RFP in tandem. When the autophagosome fuses with a lysosome to form an autolysosome, the GFP signal is quenched due to its sensitivity to the lysosomal pH. Hence, autophagosomes are observed as yellow puncta due to the co-expression of GFP and RFP, whereas autolysosomes are red. To assess autophagic flux, palmitic acid was used as a positive control to induce autophagy [37]. The images obtained showed a co-localization of the fluorescence of GFP and RFP in the untreated cells, while there was an accumulation of red vesicles due to RFP expression in the cells treated with palmitic acid or HCA (Pearson’s correlation coefficient: 0.57 ± 0.06, untreated; 0.21 ± 0.06, palmitic acid; 0.24 ± 0.04, HCA: Figure 3D). Finally, HCA did not alter the activity of the Cathepsin B enzyme that is involved in the proteolysis associated with autophagy, which therefore remained functional to be able to degrade the content of lysosomes (Supplementary Figure S3C).

### 3.4. Activation of the JNK/c-Jun/CHOP Pathway Is Related to the Mechanism of Action of HCA

**Figure 4:**
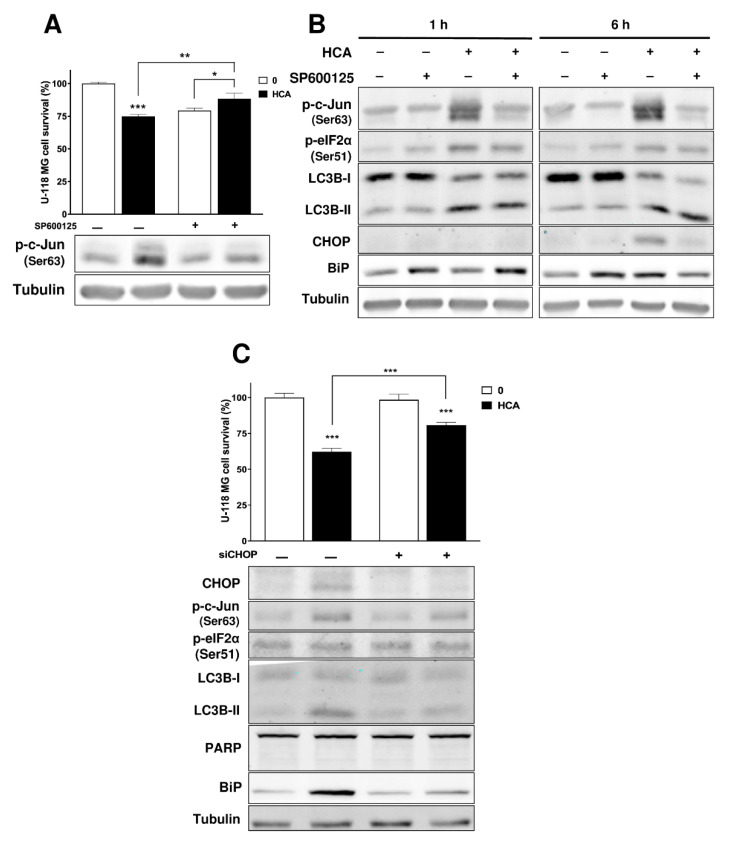
The JNK/c-Jun/CHOP pathway is related to the mechanism of action of HCA in U-118 MG GBM cells. (**A**) Effect of SP600125 on the survival of cells treated for 48 h with HCA (the bars correspond to the mean ± SEM values of 3 independent experiments performed in triplicate). (**B**) The effect of SP600125 on p-c-Jun (Ser63) levels of cells treated with HCA for 1 and 6 h. U-118 MG cells were pretreated with SP600125 for 1.5 h before exposure to HCA (150 µM). (**C**) U-118 MG cells transiently transfected with siRNA against CHOP for 6 h, after exposure to HCA (200 μM) for 24 h, or both (the bars correspond to the mean ± SEM values of 3 independent experiments performed in triplicate). Student’s t-test: *** *p* < 0.001, ** *p* < 0.01, * *p* < 0.05. HCA: 2-hydroxycervonic acid; SP600125: JNK (C-Jun N-Terminal Kinase 1) inhibitor; BIP: a.k.a. GRP78, glucose-regulated protein 78; CHOP: a.k.a. DDIT3, DNA Damage Inducible Transcript 3; c-Jun: Jun Proto-Oncogene, AP-1 Transcription Factor Subunit; eIF2a: Eukaryotic Translation Initiation Factor 2A; LC3B: a.k.a. ATG8F, Microtubule Associated Protein 1 Light Chain 3 Beta; PARP: Poly(ADP-Ribose) Polymerase; +: presence of the drug indicated in the figure; –: absence of the drug indicated in the figure.

The phosphorylation of c-Jun is one of the first events to occur in the cell after only one hour of exposure to HCA. To evaluate whether c-Jun phosphorylation was involved in the mechanism of action of HCA, the JNK inhibitor SP600125 [38] was used to inhibit the phosphorylation of c-Jun by this kinase. SP600125 inhibited the HCA antiproliferative effect in U-118 MG cells (20.7 ± 1.9% cell death in SP600125-treated cells vs. 11.6 ± 4.3% cell death in SP600125 and HCA-treated cells) (Figure 4A).

The effect of this JNK inhibitor combined with HCA on different proteins was also studied, and this combined treatment did not reverse the increase in eIF2α phosphorylation or LC3B levels induced by HCA, yet it did prevent the rise in CHOP and BiP (Figure 4B). Hence, p-c-Jun activation by JNK appeared to be necessary to activate CHOP and BiP. The impact of the CHOP protein on the mechanism of action of HCA was also studied by examining the effect of RNA interference to silence CHOP mRNA expression. The use of siCHOP largely inhibited the anti-proliferative effect of HCA in U-118 MG cells (37.8 ± 2.3% cell death in HCA-treated cells vs. 19.2 ± 2% cell death in siCHOP- and HCA-treated cells: Figure 4C). Moreover, CHOP silencing inhibited the increase in BiP and the accumulation of LC3B-II induced by HCA. Thus, these results appear to suggest that c-Jun and CHOP are critical to the mechanism of action of HCA.

### 3.5. HCA Is Incorporated into GBM Cells and It Is Associated with the Appearance of Heneicosapentaenoic Acid (C21:5n-3)

**Figure 5:**
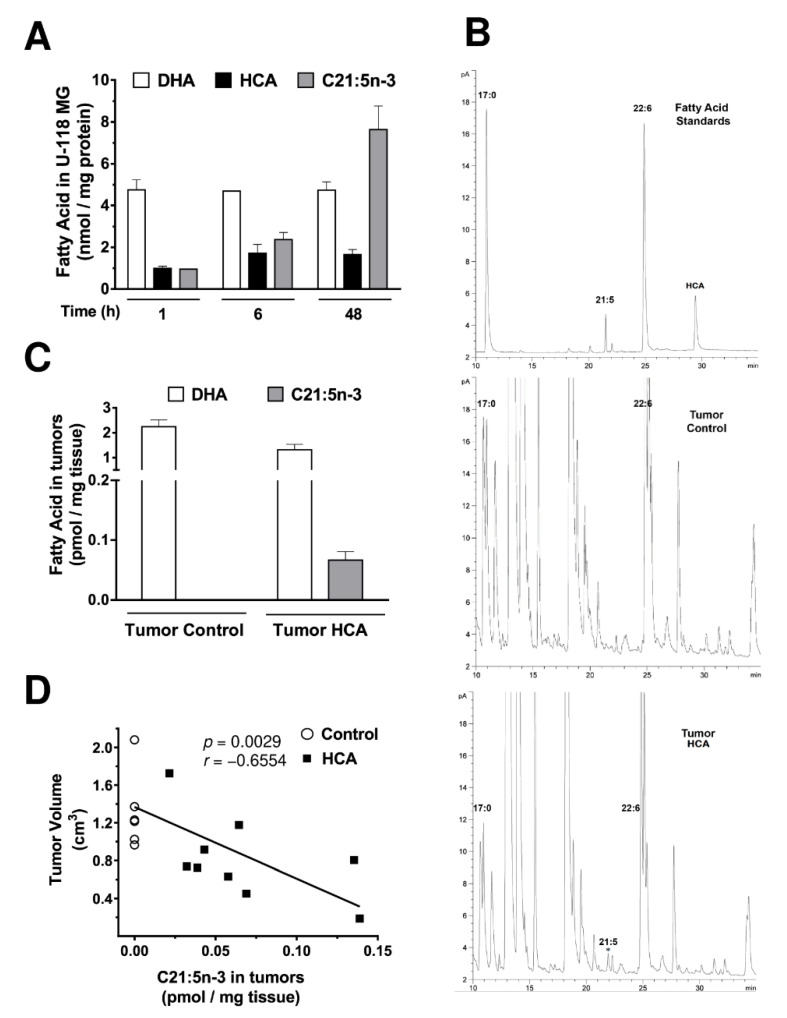
Effect of HCA on the fatty acid composition of U-118 MG cells. (**A**) U-118 MG cells were maintained in the presence or absence of HCA (150 µM) for 1, 6, or 48 h, and the lipids were then extracted from the cells. Fatty acid levels (DHA, HCA, and C21:5n-3) were quantified by GC and identified with standards (bars correspond to the mean ± SEM values of at least 3 independent experiments). (**B**,**C**) Fatty acid levels (DHA, HCA, and C21:5n-3) were identified by comparison with standards (Standards in the upper panel, Tumor Control in the second panel, and HCA-Treated Tumor in the third panel) and quantified by GC in xenograft tumors (bars correspond to the mean ± SEM values of 7–9 tumors). (**D**) Correlation between the amount of the C21:5n-3 metabolite in tumors and the volume of the tumors. HCA: 2-hydroxycervonic acid; DHA: docosahexaenoic acid; C21:5n-3: heneicosapentaenoic acid; *p* = *p*-value; *r* = Pearson’s correlation coefficient.

On exposure to HCA, the incorporation of HCA into the GBM cell lines was confirmed by gas chromatography (GC, Figure 5A, Supplementary Figure S4), whereas unsurprisingly, there was no HCA detected in control cells not exposed to HCA or in those exposed to DHA (Figure 5A, Supplementary Figure S4A,B). However, new fatty acyl peaks were observed in the chromatograms of cells treated with HCA as well as DHA, and further studies confirmed that heneicosapentaenoic acid (C21:5n-3) also accumulated in these cells after exposure to HCA [16]. The amount of this fatty acid in these cells was dependent on the length of time they were exposed to HCA, and its presence exclusively in the cells treated with HCA suggested that it arises through HCA metabolism, as described [16].

In addition, the lipids in tumors from the U-118 MG cell xenograft model were analyzed by GC once the treatment of the mice had been completed and the tumors removed. Curiously, HCA could not be detected in the tumors recovered from the mice treated with this compound. However, and somewhat surprisingly, its metabolite, C21:5n-3, was detected and could be quantified (0.06 ± 0.01 pmol/mg tissue) in the tumors recovered from the mice that were treated with HCA (Figure 5B,C). The correlation between the amount of C21:5n-3 in tumors and the volume of tumors was also studied, revealing a negative correlation between the amount of metabolite present in the tumors and the tumor volume (Pearson’s correlation coefficient, *r* = −0.6554, *p*-value = 0.0029: Figure 5D). Hence, the more this metabolite was detected in the tumor, the smaller its volume, indicating a specific antitumor effect of HCA or its metabolite.

### 3.6. The Anti-Proliferative Effect of C21:5n-3

**Figure 6:**
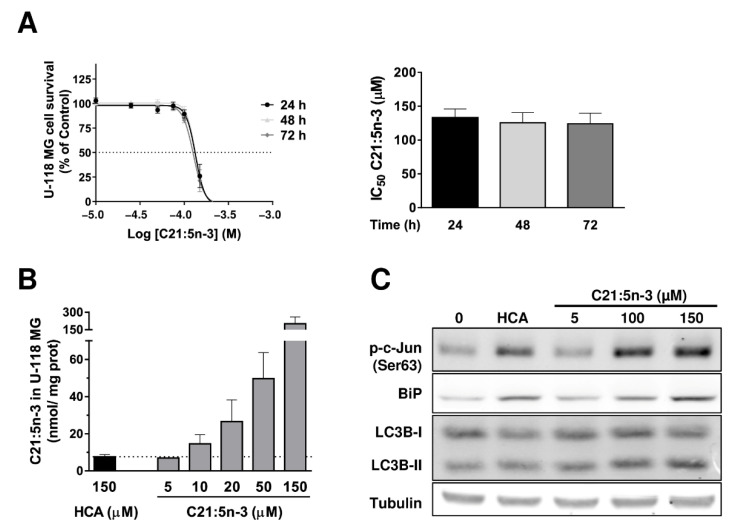
Study of the C21:5n-3 metabolite in U-118 MG cells. (**A**) Time- and concentration-dependent inhibition of U-118 MG cell growth by C21:5n-3 (left) and IC_50_ values for C21:5n-3 against U-118 cells’ proliferation after 24, 48, and 72 h treatments (mean ± SEM from 3 independent experiments performed in quadruplets, right). (**B**) Comparison of the amount of C21:5n-3 in cells treated for 48 h with C21:5n-3 (5, 10, 20, 50, and 150 µM) or HCA (150 µM) and analyzed by GC (the bars correspond to the mean ± SEM of 2 independent experiments). (**C**) Representative immunoblots of the effect of C21:5n-3 (5, 100, or 150 µM for 48 h) on ER stress/UPR and autophagy markers in U-118 MG cells. HCA: 2-hydroxycervonic acid; C21:5n-3: heneicosapentaenoic acid; BIP: a.k.a. GRP78, glucose-regulated protein 78; c-Jun: Jun Proto-Oncogene, AP-1 Transcription Factor Subunit; LC3B: a.k.a. ATG8F, Microtubule Associated Protein 1 Light Chain 3 Beta.

To determine if the C21:5n-3 metabolite influences proliferation, a cell viability test was performed on U-118 MG cells in which C21:5n-3 induced a concentration-dependent inhibition of cell growth (Figure 6A). These cells showed standard growth dynamics in the absence of C21:5n-3, yet exposure to concentrations between 100 and 150 µM of C21:5n-3 induced GBM cell death. Not only was a reduction in GBM cell number observed at all the times studied, but similar IC_50_ values were obtained for C21:5n-3 in U-118 MG cells at each of these time points: 24 h, 132.7 ± 13.3 µM; 48 h, 125.7 ± 15 µM; 72 h, 124 ± 15.7 µM (Figure 6A). The incorporation of C21:5n-3 into U-118 MG cells when they were exposed to this metabolite was studied, and these cells accumulated the same amount of this metabolite when treated with 5 µM of C21:5n-3 for 48 h (6.9 ± 0.2 nmol/mg protein) as when they were exposed to 150 µM of HCA for 48 h (7.6 ± 3 nmol/mg protein: Figure 6B). Moreover, exposure to C21:5n-3 produced an increase in p-c-Jun phosphorylation and an increase in BiP in U-118 MG cells, as well as an increase in LC3B-II protein, similar to that produced upon exposure to HCA (Figure 6C). Therefore, C21:5n-3 also appears to activate UPR and autophagic flux in U-118 MG cells.

### 3.7. Metabolization of HCA to C21:5n-3 Is Produced through α-Oxidation

**Figure 7:**
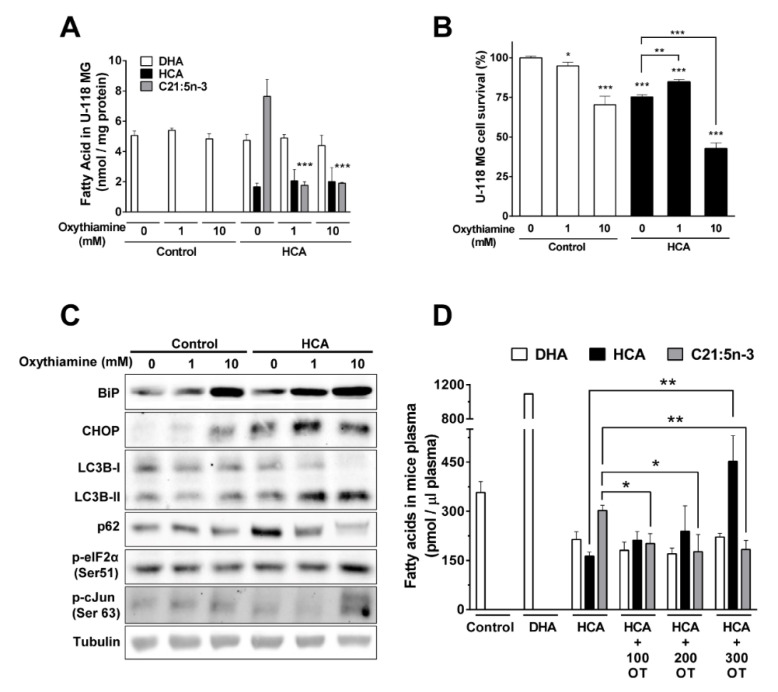
The α-oxidation of HCA to C21:5n-3. (**A**) U-118 MG cells were pretreated with oxythiamine (OT, 1 or 100 mM) for 1.5 h before exposure to HCA (150 µM) for 48 h, and the cell’s lipids were extracted and quantified by GC (all bars correspond to the mean ± SEM values of at least 3 independent experiments). (**B**) Effect of OT on the survival of cells treated for 48 h with HCA (bars correspond to the mean ± SEM values of 3 independent experiments performed in triplicate), and (**C**) representative immunoblots of the effect of OT and HCA on ER stress/UPR and autophagy markers in U-118 MG cells. (**D**) The quantity of fatty acids in the plasma of mice treated with OT (100, 200, or 300 mg/ kg, p.o., daily) and HCA (200 mg/kg, p.o., daily) for 7 days (the bars correspond to the mean ± SEM values of at least 3 plasma mice samples). Student’s t-test: *** *p* < 0.001, ** *p* < 0.01, * *p* < 0.05. HCA: 2-hydroxycervonic acid; DHA: docosahexaenoic acid; C21:5n-3: heneicosapentaenoic acid; OT: oxythiamine; BIP: a.k.a. GRP78, glucose-regulated protein 78; CHOP: a.k.a. DDIT3, DNA Damage Inducible Transcript 3; c-Jun: Jun Proto-Oncogene, AP-1 Transcription Factor Subunit; eIF2a: Eukaryotic Translation Initiation Factor 2A; LC3B: a.k.a. ATG8F, Microtubule Associated Protein 1 Light Chain 3 Beta; p62: a.k.a. SQSTM1, Sequestosome 1.

Fatty acids hydroxylated at the alpha-carbon can be metabolized by α-oxidation [39]. This metabolic route is mediated by a hydroxy-acyl CoA lyase (HACL1 or 2, depending on the cell type), which in turn is dependent on thiamine pyrophosphate (TPP), so the oxythiamine (OT), thiamine analog, and an inhibitor of HACL1, was used to confirm that the C21:5n-3 was produced from HCA α-oxidation [16]. When U-118 MG cells were maintained in the presence of OT prior to their exposure to HCA (150 µM) for 48 h, there was a significant 76% reduction in the C21:5n-3 metabolite (from 7.6 ± 11 to 1.8 ± 0.2 nmol/mg prot) identified by GC among the fatty acids from these cells, both with 1 or 10 mM of OT (Figure 7A). In contrast, the amount of HCA detected in these cells remained constant. Since OT inhibited the formation of C21:5n-3 via α-oxidation in cells exposed to HCA to a large extent, its effect on cell survival and the alterations in protein accumulation provoked by HCA was studied. OT alone provoked a significant concentration-dependent decrease in U-118 MG cell survival (95 ± 2.3% survival with 1 mM OT, 70.3 ± 5.4% survival for 10 mM OT: Figure 7B), in accordance with the antitumor effect of OT previously described in vitro and in vivo [40]. However, while the combination of OT (1 mM) with HCA (150 µM) appeared to enhance cell viability at 48 h (84.8 ± 1.5% survival) relative to the cells that were exposed to HCA alone (75.2 ± 1.5%), when administered at 10 mM, OT clearly exacerbated HCA-provoked cell death (42.6 ± 3.7% survival), reflecting a synergistic effect of the two compounds. This synergism between OT and HCA was also evident in the potentiation of the effects of HCA on proteins involved in different signaling pathways and cell death, exacerbating the increase in BiP, CHOP, and LC3B-II, and further augmenting the increase in c-Jun and eIF2α phosphorylation (Figure 7C). Finally, the effect of OT (100, 200, or 300 mg/kg, p.o., daily), HCA (200 mg/kg, p.o., daily), a combination of both, and DHA (200 mg/kg, p.o. daily) in vivo was studied in mouse plasma. Mice were administered these compounds for 7 days, and the fatty acids in the plasma extracted were analyzed by GC. The amount of C21:5n-3 detected in the plasma of mice administered HCA alone (302.4 ± 15.49 pmol/µl plasma) was significantly higher than that in mice treated with both HCA and OT (201.2 ± 30.63 pmol/µl plasma for 100 mg/kg OT, 176.6 ± 52.24 pmol/µl plasma for 200 mg/kg OT, 183.4 ± 27.4 pmol/µl plasma for 300 mg/kg OT: Figure 7D). Moreover, in the plasma recovered from the mice administered both compounds, there was significantly more HCA detected (from 163.1 ± 12.29 pmol/µl plasma for only HCA treatment to 211 ± 27.02 pmol/µl plasma for 100 mg/kg OT, 238.9 ± 77.82 pmol/µl plasma for 200 mg/kg OT, and 451.8 ± 78.55 pmol/µl plasma for 300 mg/kg OT in combination with HCA treatment), even though no significant differences in the amounts of C21:5n-3 were recovered at the 3 doses of OT administered (Figure 7D). These data clearly show that OT is capable of reducing the formation of C21:5n-3 from HCA in vivo.

## 4. Discussion

The current standard-of-care treatment for GBM involves a combination of radiotherapy and chemotherapy with TMZ [4], yet this treatment only increases the average survival of patients by 10 weeks, to reach a median survival of 14.5 months, reflecting the urgent need to develop new drugs that improve the outcome of this pathology. We tested the potential for a rationally designed compound derived from DHA to halt the progression of GBM, the omega-3 fatty acid HCA that is modified by the addition of a hydroxyl moiety to the alpha-carbon of DHA. The data obtained suggest that this compound potentially limits the growth of GBM tumor cells and that it even provokes the death of these cells. In light of the obtained data, it would clearly be worthwhile to undertake more clinically orientated studies to further assess the potential of HCA to combat GBM in patients.

Hydroxylated fatty acids can be found in nature, such as resolvins and protectins, formed from the omega-3 fatty acids EPA and DHA, and these compounds have been shown to offer neuroprotection and to have anti-inflammatory properties in various pathologies [41]. Furthermore, resolvins suppress tumor growth and enhance cancer therapy [42]. These clinically relevant pharmaceutical activities of natural hydroxylated fatty acids encouraged us to produce HCA and test its properties [43]. The design of HCA was encompassed in the sphere of melitherapy, a therapeutic approach that regulates the composition and structure of cell membranes as a means to treat different pathologies [6,44]. One example of a compound based on this approach is 2OHOA, a hydroxylated derivative of oleic acid that has an important potential in combatting different types of cancer [45]. This compound has successfully completed a phase I/IIA clinical trial in patients with advanced gliomas and other solid tumors [7] (clinicaltrial.gov, NCT01792310), showing therapeutic benefits in almost half of the GBM patients who were refractory to other treatments. Based on this study, the Maximum Tolerated Dose (MTD) was established at 16 g/day, and the recommended therapeutic dose was fixed at 12 g/day, showing a high safety profile and allowing to reach high concentrations to ensure the antitumor activity. As a result, a pivotal phase IIB study in patients with GBM has been initiated to further demonstrate the efficacy and safety of 2OHOA. Thus, these clinical studies provide evidence of the potential of hydroxylated lipids to join the armory in the fight against cancer, encouraging the search for more potent and specific derivatives of these therapies. One of the advantages of the molecules working through the melitherapy is the safety that they have demonstrated in different models, indicating that they can have a large therapeutic window to treat diverse pathologies and reach high doses (including the doses tested in this work in the animal models) without showing any adverse side effects [7,17,46].

In this study, the efficacy of the synthetic fatty acid HCA in the treatment of glioma was evaluated, demonstrating that HCA has a negative effect on the proliferation of GBM cells, inducing cell death in a time- and concentration-dependent manner. In cell viability studies, HCA was attributed an IC_50_ between 100 and 200 µM. Regarding TMZ, it was observed that U-118 MG cells incubated with TMZ (250 µM) inhibited their proliferation rate by 43.2% with respect to control cells [47]. As a first proof-of-concept, HCA anti-tumor efficacy in vivo was confirmed in a xenograft model of glioma tumors in immunosuppressed mice, which allows to assess the impairment of the tumor growth by measuring the tumor during the treatment, even if the environment and the location are not the ones that could be expected for a GBM in humans. HCA has a strong anti-tumor effect in vivo, reducing the volume of tumors by 45% on average and even making tumors disappear in some animals. Although TMZ is more effective than HCA in reducing tumor size, producing a 67% reduction in size on average, none of the tumors had a complete reversion, and its activity is associated with several important side effects, including reversible myelosuppression and thrombocytopenia [48], tumor recurrence, and the development of resistance (at least 50% of patients fail to respond to TMZ) [5]. In addition to its efficacy against GBM cells, HCA was seen to be very safe in vivo in studies on zebrafish, *D. melanogaster,* and mice [8,15,32]. Indeed, the immunosuppressed mice used here did not lose more than 10% of their weight when treated with HCA for more than 40 days. Thus, in conjunction with the data obtained here, it suggests that it would be interesting to further study the consequences of different regimens of HCA administration in vivo in order to evaluate and optimize the effect of this drug alone, and when combined with TMZ therapy, in a xenograft immunosuppressed mice model together with orthotopic and/or spontaneous genetic ones. HCA activates the different pathways of UPR and autophagy in GBM cells, reminiscent of its modulation of UPR and autophagy in differentiated neuron-like SH-SY5Y cells [17]. HCA augments the levels of BiP, those of proteins in the eIF2α/ATF4/CHOP and IRE/c-Jun pathways, and of ATF6. When physiological and pathological conditions provoke ER stress in cells, the UPR is activated to re-establish ER homeostasis. However, when ER stress persists, the protective functions of the UPR and of autophagy shift towards the induction of cell death [49]. Recently, a variety of cancer therapies have been linked to the induction of ER stress in cancer cells, devising strategies that favor cell death or block survival strategies to improve the anti-tumor activity of certain drugs [49]. For instance, the drug 2OHOA induces cell death by autophagy [26], whereas the autophagy induced by TMZ in some GBM cell lines may produce resistance to treatment and suppress its anti-tumor effects [50]. In terms of the regulation of autophagy by HCA, the increase in autophagic flux was indicated by the conversion of LC3B-I to LC3B-II. LC3B is a protein encoded by autophagy-related gene 8 (ATG8) and is modified by an addition of a phosphatidylethanolamine (LC3B-II) during the formation of the autophagolysosomes [22,34]. The ATG proteins are a family of proteins that are activated during the autophagy induction and coordinate the different steps of the process until the final digestion of the content into the autophagolysosomes. There was also an increase in the sequestosome protein p62 that links ubiquitinated proteins to the autophagic machinery in order to drive their degradation in the lysosome. The accumulation of p62 to recruit the ubiquitylated cargo proteins could be a delayed event with respect to LC3B-II formation and be maintained over a long period of time until all the proteins are degraded, as described previously [21]. Additional experiments demonstrated that autophagy was completed by the degradation of cell components in the lysosome of cells treated with HCA. First, treating cells with HCA and BafA1 enhances autophagic flux, the latter being a compound that blocks the fusion between autophagosomes and lysosomes. Second, monitoring autophagic flux by transfection with the ptfLC3 plasmid confirmed that it was enhanced by HCA and culminated in the degradation of cell components in the lysosomes. Combination exposure to HCA with that of a PI3K class III inhibitor (3-MA) recovers cell survival, confirming that cell death is induced by HCA via autophagy. Finally, the activity of Cathepsin B in lysosomes is not altered by HCA, and this enzyme maintains its capacity to degrade cell components.

HCA triggers a rapid stress response in glioma cells, increasing the amounts of proteins such as p-c-Jun, p-eIFα, and LC3B in as little as one hour after exposure, one of the first events induced by HCA. Activation of the c-Jun pathway appeared to be critical for the effect of HCA on cell survival and to subsequently activate the CHOP and BiP proteins. Hence, the JNK/c-Jun pathway appears to be a trigger for cell stress, and consequently, an inducer of death by autophagy. JNK is known to regulate autophagy at multiple levels, involving both nuclear and cytoplasmic events. In the nucleus, JNK increases the expression of several autophagy-related genes, such as ATGs and the FoxO transcription factors [51]. Moreover, in relation to the cell death induced by cisplatin, recent evidence suggests that JNK signaling plays an important role in its mechanism of action and, in particular, in the decision of whether or not to induce resistance to apoptosis by activating autophagy [52]. HCA does not induce cell death in the absence of CHOP (i.e., after silencing its expression through an RNAi), indicating that this protein is critical for this effect of HCA. In addition, the loss of CHOP meant that cells did not augment their BiP and LC3B-II in response to HCA. CHOP is a multifunctional transcription factor that contributes to various cellular activities, including apoptosis, autophagy, inflammation, cell differentiation, and proliferation [53]. Various antitumor compounds activate CHOP to induce death by autophagy or apoptosis. For example, sulfonamide benzamide and isochaihulactone are CHOP inducers with pro-apoptotic and anti-proliferative effects on multiple cancer cell lines [54]. In summary, the data obtained with inhibitors and HCA indicate that the JNK/c-Jun/CHOP/BiP axis appears to regulate cell death by autophagy activated by HCA.

HCA is a lipid that was designed based on the knowledge that fatty acids are incorporated into cells and that they can modulate the lipid composition of membranes. It was first confirmed that HCA incorporates into the tumor cell to induce cell death, comparing its incorporation with that of DHA. Curiously, less HCA was incorporated by the cell than DHA, as seen elsewhere [16], which may be due to the presence of the hydroxyl group of the alpha-carbon of HCA, making its entry into the lipid bilayer more difficult [16]. In addition, a new metabolite appeared in the cells incubated with HCA in a time- and dose-dependent manner, heneicosapentaenoic acid (C21:5n-3). This metabolite only appears following exposure to HCA, suggesting that it is a specific active pharmacological metabolite of HCA, as further supported by the fact that its levels were systematically higher than those of HCA in the treated cell cultures. Furthermore, it has been observed that HCA administration in mice leads to elevated levels of C21:5n-3 in blood plasma and the brain [16], as well as in the remaining tumors after treatment. The detection of the metabolite in the brain and in the tumors supports the potential ability of HCA to cross the blood–brain barrier to reach the brain and act as a drug against GBM, directly or after its metabolization as C21:5n-3. Since HCA is metabolized by α-oxidation and both molecules had a similar pharmacokinetic profile [16], OT was used to inhibit HACL1, a key enzyme in α-oxidation [39], indicating that α-oxidation of HCA is required to produce this C21:5n-3 metabolite. Moreover, administering HCA and OT to mice also appeared to inhibit C21:5n-3 formation from HCA in vivo. However, the inhibition of metabolite formation was incomplete even after incubation with the highest dose of OT, so that low levels of C21:5n-3 were detected after HCA administration under all conditions. Furthermore, OT itself has an anti-proliferative effect on tumor cells at a relatively high concentration (10 mM), as described previously in vitro and in vivo [40], in part due to the inhibition of the transketolase (a key enzyme in the pentose-phosphate pathway [55]). Although, OT use here also demonstrated the need for HCA to be metabolized to C21:5n-3 in order to exert its anti-proliferative effects. Interestingly, the lower dose of OT (1 mM) used here partially inhibited the anti-proliferative effects of HCA, whereas it had a synergistic effect with HCA at the higher dose (10 mM), enhancing the reduction in cell proliferation induced by HCA, as well as the ER stress in the cells and their death. This synergistic or combined effect could be due to the toxicity induced by the high dose of OT used as well as the antitumor effect of OT by itself, together with the effect of the remaining C21:5n-3 present in the cells or a possible mechanism triggered by HCA in a metabolite-independent manner (considering that the inhibition of the cell death induced by HCA upon OT incubation was incomplete).

Once taken up, a drug may be metabolized inside the body to inactive products or to compounds that have a therapeutic action or that are toxic. For example, TMZ is a DNA alkylating agent that is considered a prodrug, i.e., a compound that needs to be metabolized in vivo after its administration in order to become active [56]. C21:5n-3 is naturally found at low concentrations in fish oil and in algae (*B. pennata*, [57]), and it has been isolated from human plasma at low concentrations [58]. C21:5n-3 is present in the diet in only trace amounts, and thus, it could be used as a biomarker to monitor HCA treatment. The presence of C21:5n-3 in the xenograft tumors of mice treated with HCA and not in xenograft tumors of control mice could confirm the specificity of this metabolite as a biomarker to follow HCA treatment. Furthermore, there was a correlation between the amount of metabolite present in tumors and their volume, whereby a smaller tumor volume was associated with the accumulation of more of this metabolite. C21:5n-3 has an anti-proliferative effect on cells, and it is more readily incorporated into the cell than HCA: after 48 h, treatment of 5 µM of C21:5n-3 is equivalent to the treatment of 150 µM of HCA in terms of their incorporation into cells. Therefore, the C21:5n-3 also seems to activate the UPR and autophagy flux. 

## 5. Conclusions

The α-hydroxylated DHA derivative HCA seems to exert an anti-proliferative effect on GBM cells by inducing ER stress and autophagy. This negative effect on GBM growth may be mediated by the JNK/c-Jun/CHOP/BiP axis and by its metabolite C21:5n-3. Further studies will be necessary to advance the clinical relevance of this data and to move towards an improved therapy for GBM (melitherapy-based), which is currently a significant unmet medical need.

## Data Availability

All data generated or analyzed during this study are included in the manuscript.

## Supplementary Materials

The following are available online at https://www.mdpi.com/article/10.3390/cancers13174290/s1, Figure S1: HCA efficacy in mice, Figure S2: HCA activates ER stress/UPR signaling pathways in SF-295 GBM cells, Figure S3: HCA induces autophagy in SF-295 GBM cells, Figure S4: Effect of HCA on the fatty acid composition of U-118 MG or SF-295 GBM cells, Figure S5: Densitometric analysis of Western blot results presented all figures, Table S1: Efficacy of HCA against different human glioma cell lines.
