# Supplementary material for: The Novel Antitumor Compound HCA Promotes Glioma Cell Death by Inducing Endoplasmic Reticulum Stress and Autophagy
Source: Cancers (Basel). 2021 Aug 26;13(17):4290. doi: 10.3390/cancers13174290 (PMC8428344; doi:10.3390/cancers13174290)
Supplement: Supplementary file 1 [file cancers-13-04290-s001.zip › cancers-1327629-supplementary.pdf]

cancers

SUPPLEMENTARY TABLE

Table S1. Efficacy of HCA against different human glioma cell lines. IC50 values for HCA against glioma cell proliferation after a 24, 48 and 72 h treatment (mean ± SEM from 3 independent experiments performed in quadruplet). HCA: 2-hydroxycervonic acid.

Cell line	Time (h)	IC_{50} HCA (μM)
**SF-268**	24	148 ± 7
	48	115 ± 8
	72	104 ± 10
**SNB-19**	24	175 ± 5
	48	132 ± 6
	72	130 ± 8
**SNB-75**	24	138 ± 9
	48	120 ± 10
	72	111 ± 10
**U-251 MG**	24	147 ± 4
	48	99 ± 7
	72	89 ± 10

SUPPLEMENTARY FIGURES

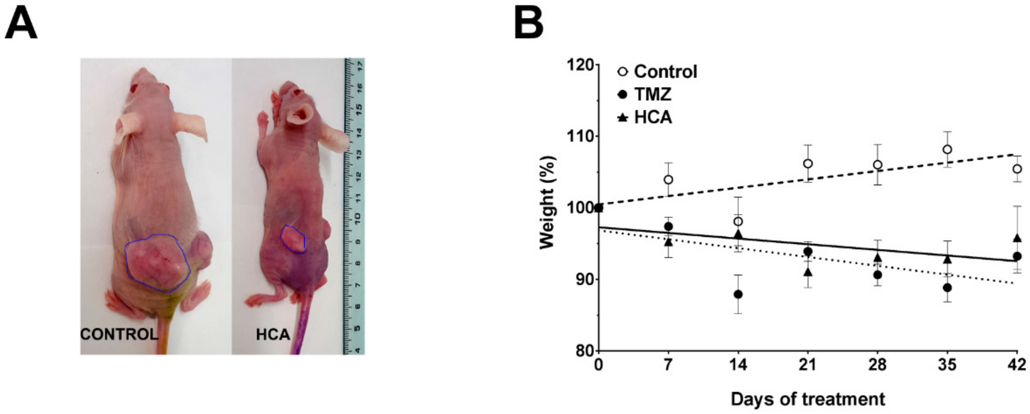

Figure S1. HCA efficacy in mice (**A**) Representative photographs of mice that received a xenograft of U-118 MG cells and treated with the vehicle alone (Control) or HCA (200 mg/kg, p.o., 42 days). (**B**) The weight of the mice was measured during the treatment (mean ± SEM: n = 22 for the controls, n = 19 for HCA, n = 8 for TMZ). HCA: 2-hydroxycervonic acid; TMZ: temozolomide.

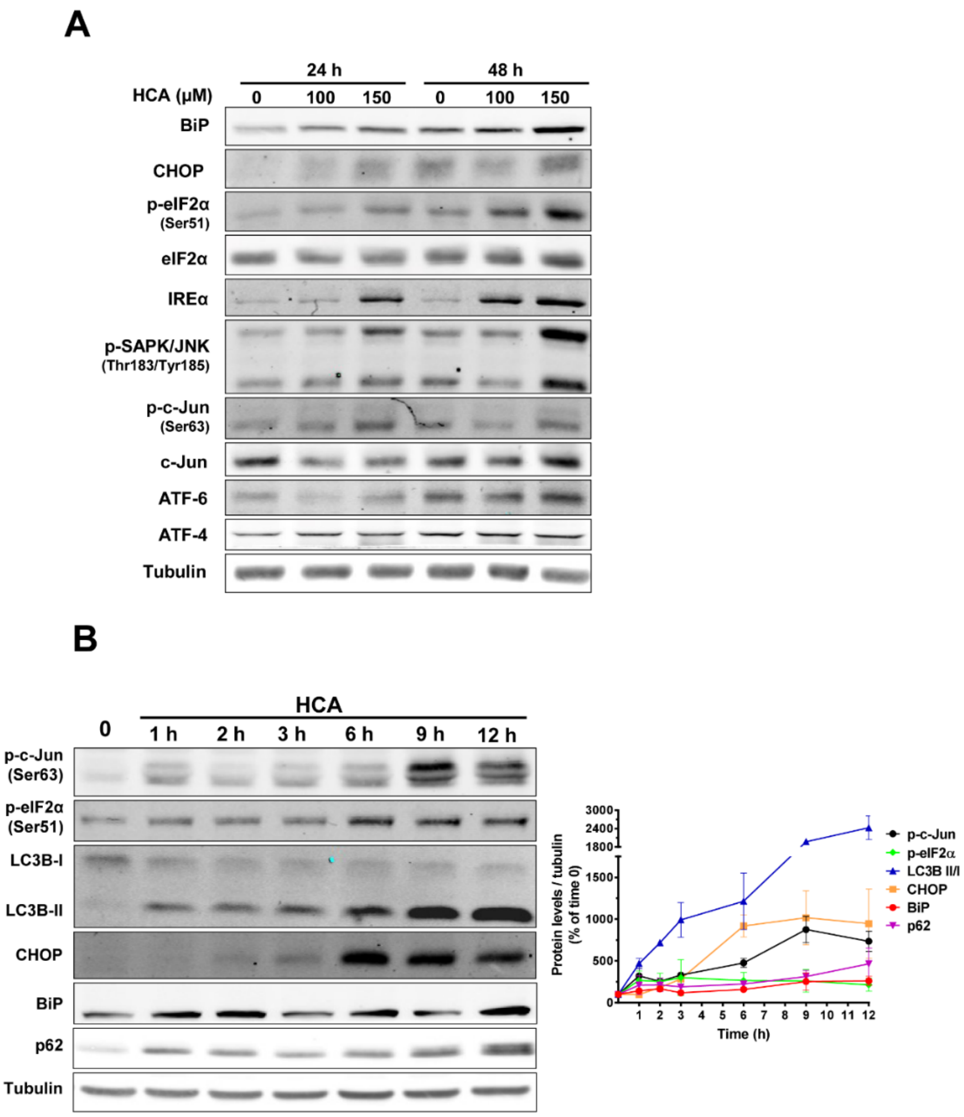

Figure S2. HCA activates ER stress/UPR signaling pathways in SF-295 GBM cells. (**A**) Representative immunoblots of the effect of HCA on SF-295 cells (100 or 150 μM for 24 or 48 h) on eIF2*α* and c-Jun phosphorylation, BiP, CHOP, ATF6, ATF4 and IRE*α* proteins, using tubulin as a loading control. (**B**) SF-295 cells were treated with 200 μM HCA for 1, 2, 3, 6, 9 and 12 h, and protein levels or phosphorylation were assessed (left) and quantified in immunoblots (mean ± SEM of three independent experiments, right). HCA: 2-hydroxycervonic acid; ATF: Activating Transcription Factor; BiP: a.k.a. GRP78, glucose-regulated protein 78; CHOP: a.k.a. DDIT3, DNA Damage Inducible Transcript 3; c-Jun: Jun Proto-Oncogene, AP-1 Transcription Factor Subunit; eIF2a: Eukaryotic Translation Initiation Factor 2A; IRE*α*: inositol-requiring enzyme 1; LC3B: a.k.a. ATG8F, Microtubule Associated Protein 1 Light Chain 3 Beta; p62: a.k.a. SQSTM1, Sequestosome 1.

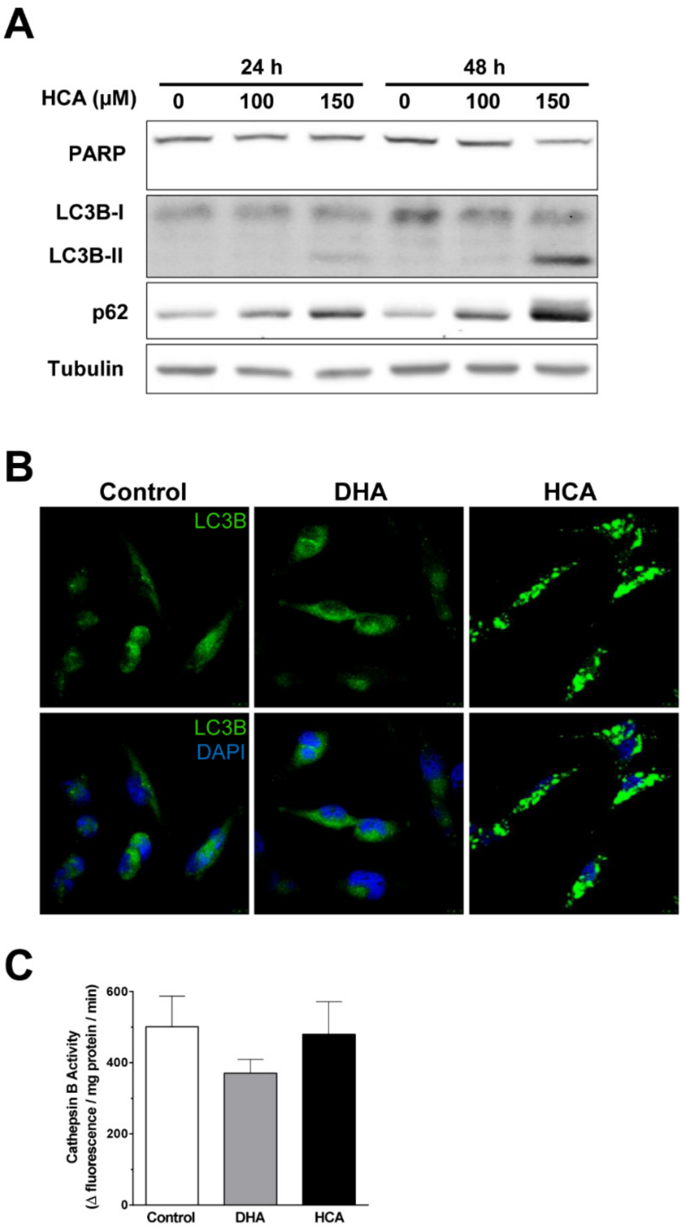

Figure S3. HCA induces autophagy in SF-295 GBM cells. (**A**) Representative immunoblots of the effect of HCA (100 or 150 µM for 24 or 48 h) on PARP, LC3B and p62 proteins in SF-295 cells, using tubulin as a loading control. (**B**) LC3B immunofluorescence (green) in SF-295 cells treated for 48 h with DHA (100 µM) or HCA (150 µM), with the nuclei labeled with DAPI. Representative micrographs (single confocal planes) are shown: scale bar, 5 µm. (**C**) Cathepsin B activity of U-118 MG cells after treatment with DHA (100 µM) or HCA (150 µM) for 48 h (bars correspond to the mean ± SEM values of 3 independent experiments). HCA: 2-hydroxycervonic acid; DHA: docosahexaenoic acid; LC3B: a.k.a. ATG8F, Microtubule Associated Protein 1 Light Chain 3 Beta; p62: a.k.a. SQSTM1, Sequestosome 1; PARP: Poly(ADP-Ribose) Polymerase.

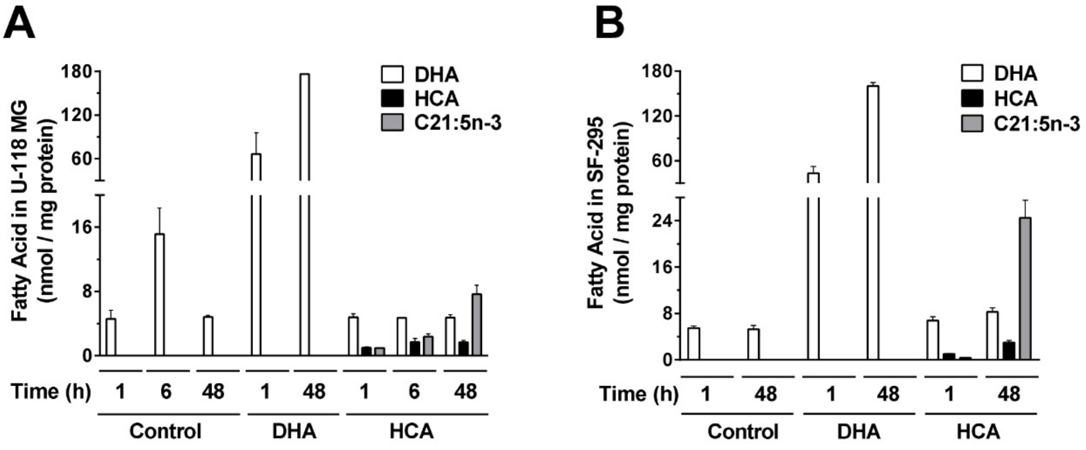

Figure S4. Effect of HCA on the fatty acid composition of U-118 MG or SF-295 GBM cells. (**A**) U-118 MG cells were maintained in the presence or absence of HCA (150 µM) or DHA (100 µM) for 1, 6 or 48 h, and the lipids were extracted from the cells. (**B**) SF-295 cells were treated as in (**A**). The levels of DHA, HCA and C21:5n-3 were quantified by GC, and identified by comparison to the standards (all the bars correspond to the mean ± SEM values of at least 3 independent experiments).Figure 2. HCA: 2-hydroxycervonic acid; DHA: docosahexaenoic acid; C21:5n-3: heneicosapentaenoic acid.

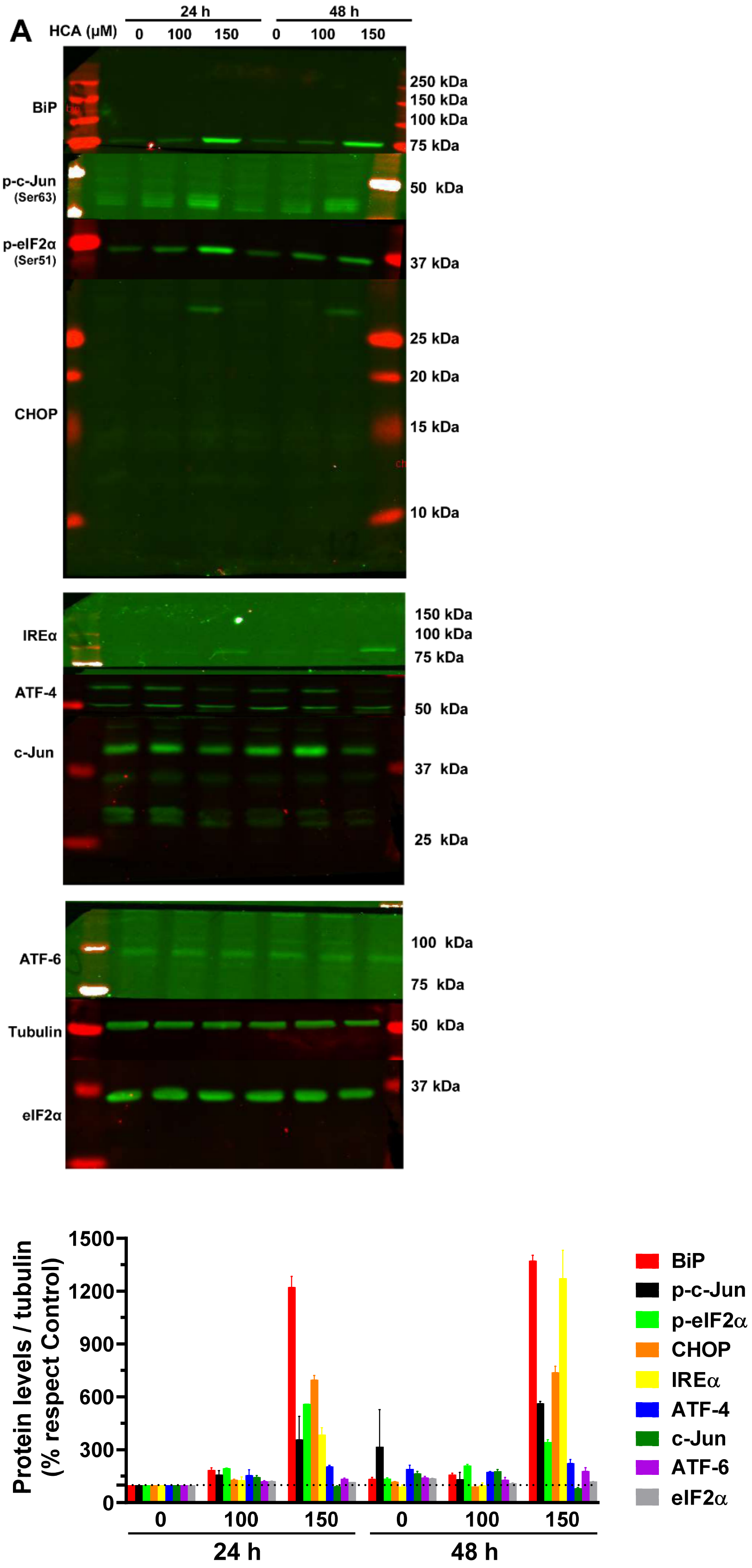
A
HCA (μM) 0 100 150 0 100 150
24 h
48 h
BiP
p-c-Jun (Ser63)
p-eIF2α (Ser51)
CHOP
IREα
ATF-4
c-Jun
ATF-6
Tubulin
eIF2α
250 kDa
150 kDa
100 kDa
75 kDa
50 kDa
37 kDa
25 kDa
20 kDa
15 kDa
10 kDa
Protein levels / tubulin (% respect Control)
1500
1200
900
600
300
0
BiP
p-c-Jun
p-eIF2α
CHOP
IREα
ATF-4
c-Jun
ATF-6
eIF2α

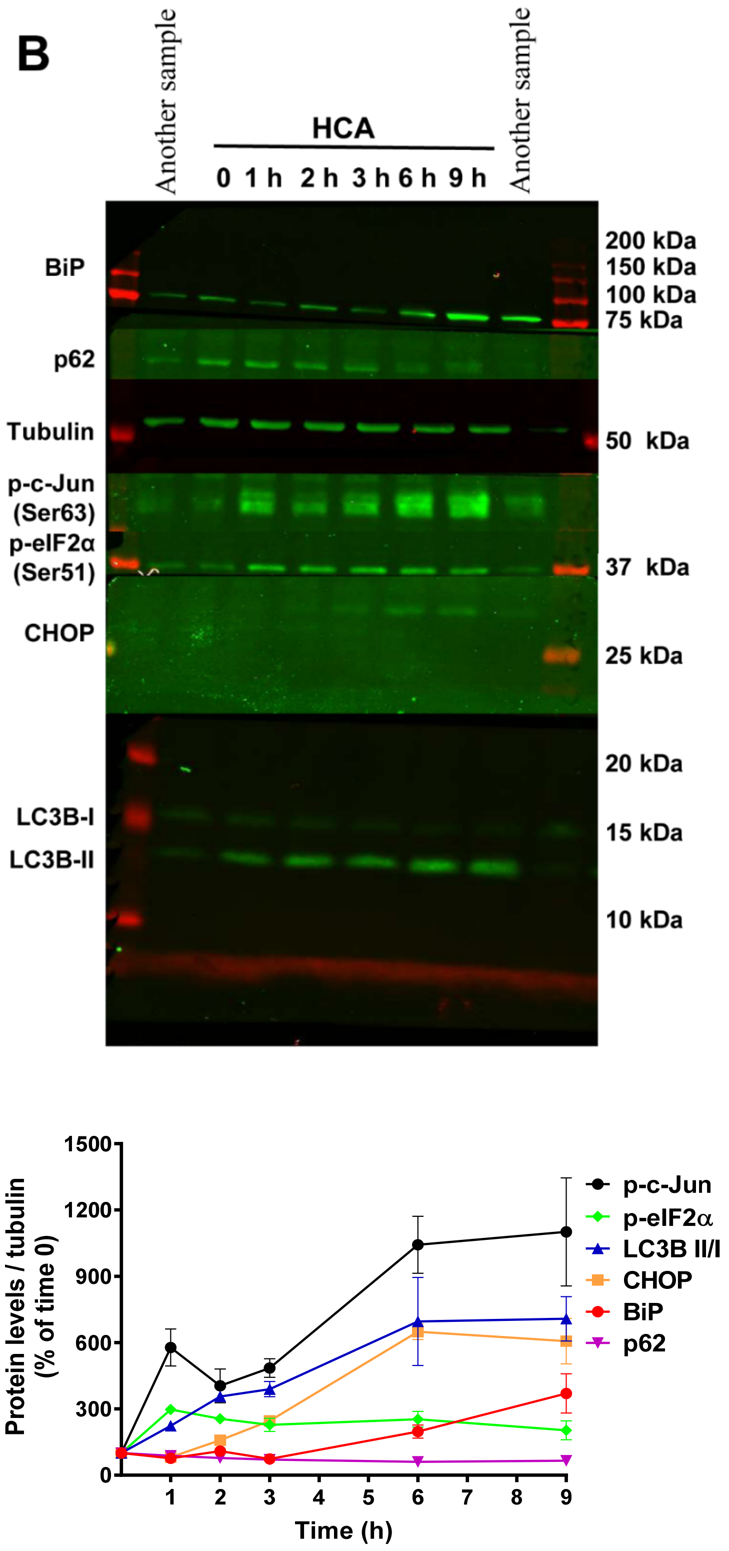

B
Another sample
HCA
0 1 h 2 h 3 h 6 h 9 h
Another sample
BiP
p62
Tubulin
p-c-Jun (Ser63)
p-eIF2α (Ser51)
CHOP
LC3B-I
LC3B-II
200 kDa
150 kDa
100 kDa
75 kDa
50 kDa
37 kDa
25 kDa
20 kDa
15 kDa
10 kDa
Protein levels / tubulin (% of time 0)
Time (h)
p-c-Jun
p-eIF2α
LC3B II/I
CHOP
BiP
p62

Figure 2.

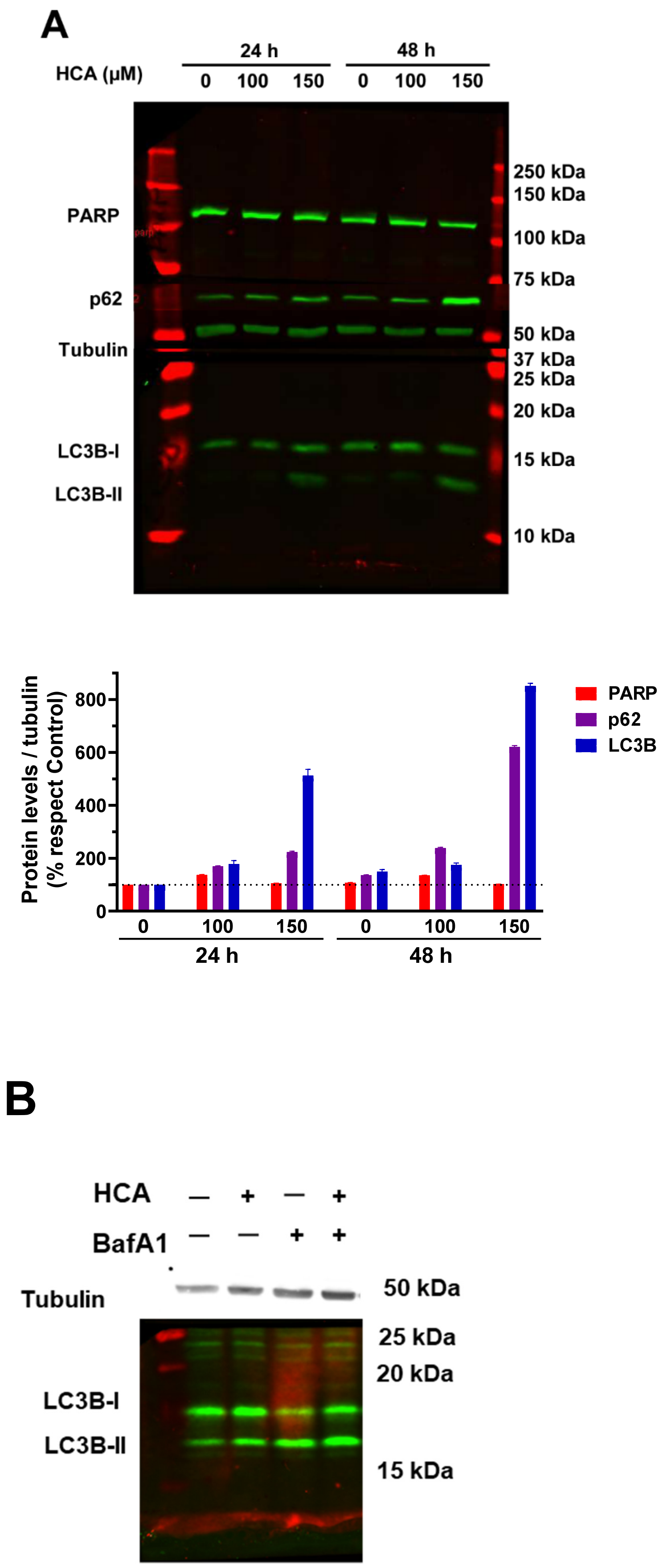
A
24 h
48 h
HCA (µM)
0 100 150 0 100 150
PARP
p62
Tubulin
LC3B-I
LC3B-II
250 kDa
150 kDa
100 kDa
75 kDa
50 kDa
37 kDa
25 kDa
20 kDa
15 kDa
10 kDa
Protein levels / tubulin
(% respect Control)
800
600
400
200
0
PARP
p62
LC3B
24 h
48 h
B
HCA
BafA1
Tubulin
50 kDa
25 kDa
20 kDa
LC3B-I
LC3B-II
15 kDa

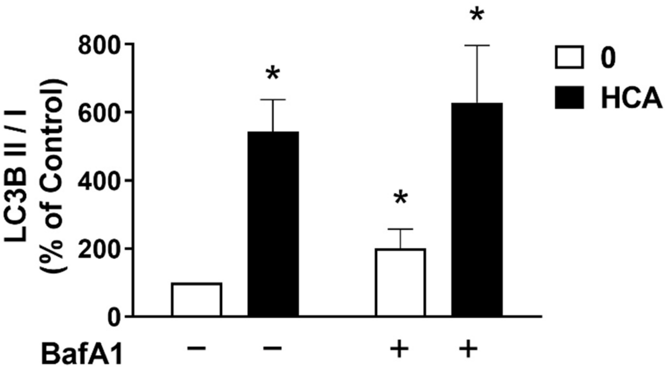
800
600
400
200
0
LC3B II / I
(% of Control)
*
*
*
0
HCA
BafA1
−
−
+
+

Figure 3.

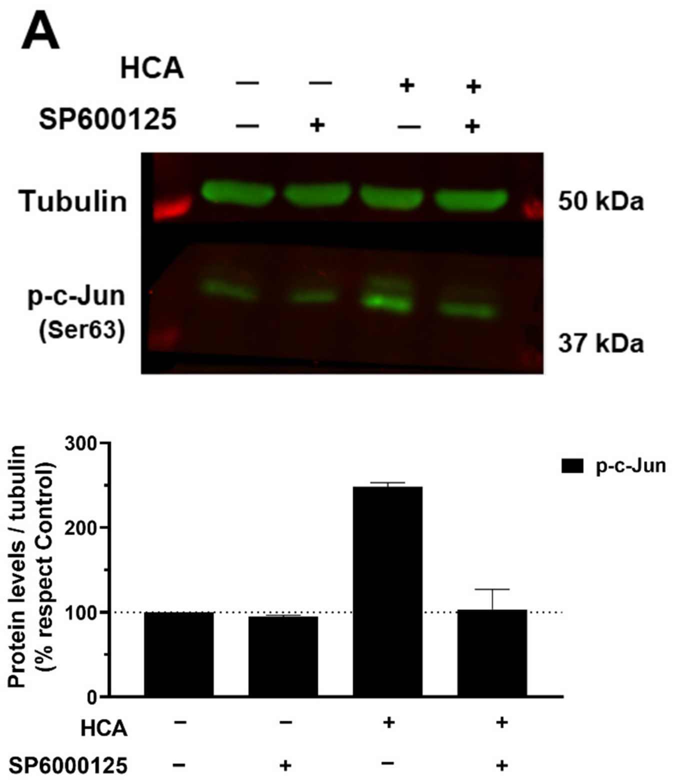
A
HCA
SP600125
Tubulin
50 kDa
p-c-Jun
(Ser63)
37 kDa
Protein levels / tubulin
(% respect Control)
300
200
100
0
p-c-Jun
HCA
SP6000125

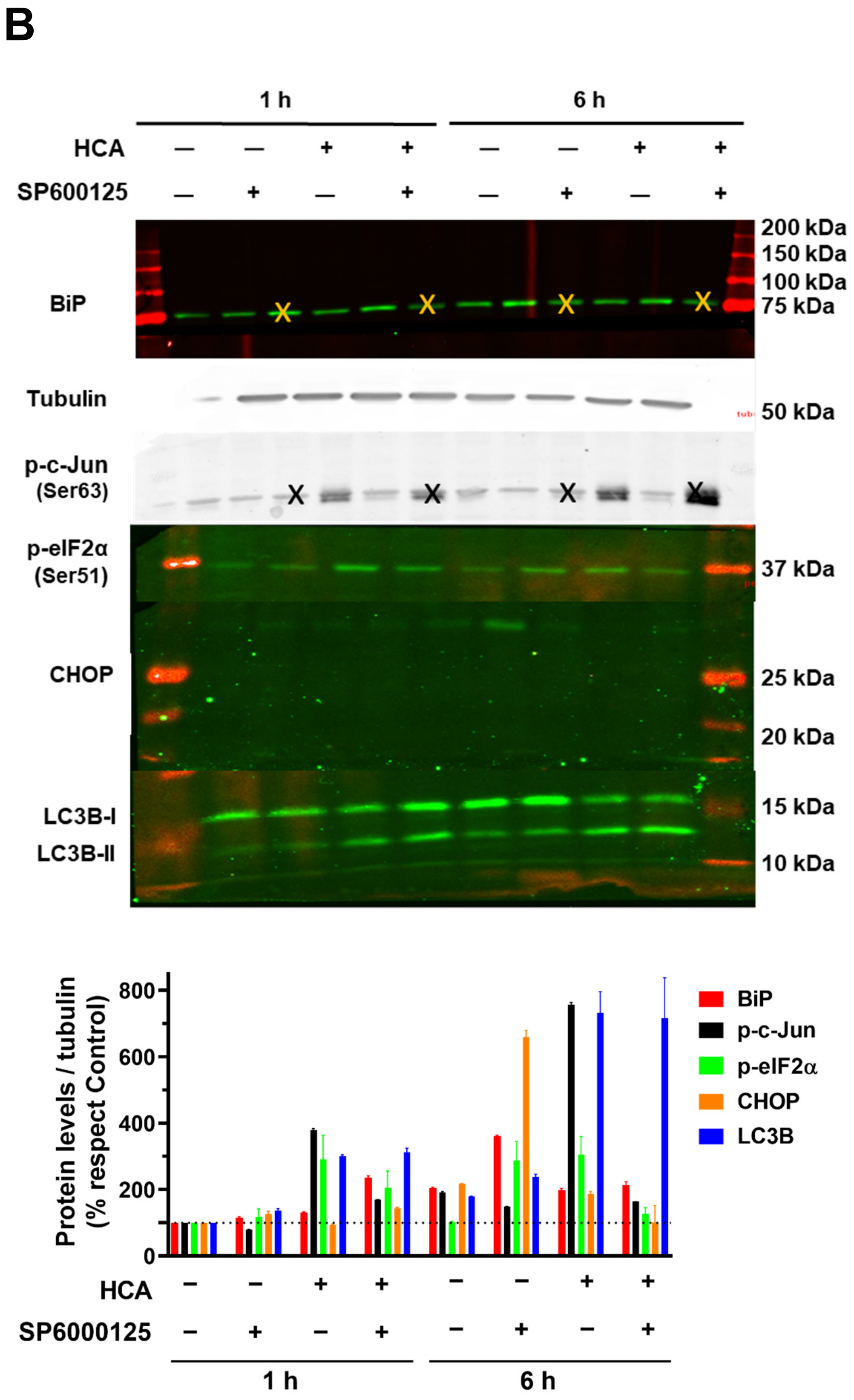
B
1 h
6 h
HCA
SP600125
BiP
Tubulin
p-c-Jun (Ser63)
p-eIF2α (Ser51)
CHOP
LC3B-I
LC3B-II
200 kDa
150 kDa
100 kDa
75 kDa
50 kDa
37 kDa
25 kDa
20 kDa
15 kDa
10 kDa
Protein levels / tubulin (% respect Control)
800
600
400
200
0
BiP
p-c-Jun
p-eIF2α
CHOP
LC3B
HCA
SP6000125
1 h
6 h

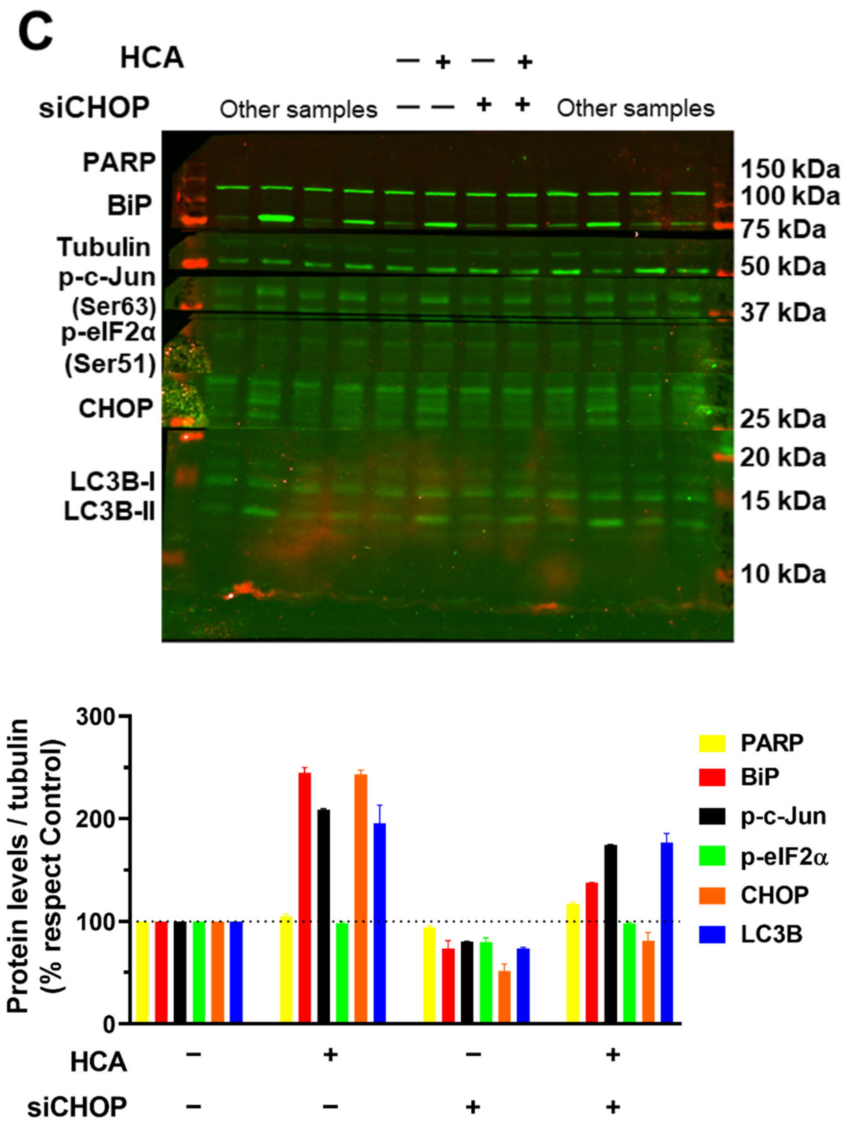
C
HCA
siCHOP
Other samples
Other samples
PARP
BiP
Tubulin
p-c-Jun
(Ser63)
p-eIF2α
(Ser51)
CHOP
LC3B-I
LC3B-II
150 kDa
100 kDa
75 kDa
50 kDa
37 kDa
25 kDa
20 kDa
15 kDa
10 kDa
Protein levels / tubulin
(% respect Control)
300
200
100
0
PARP
BiP
p-c-Jun
p-eIF2α
CHOP
LC3B
HCA
siCHOP

Figure 4.

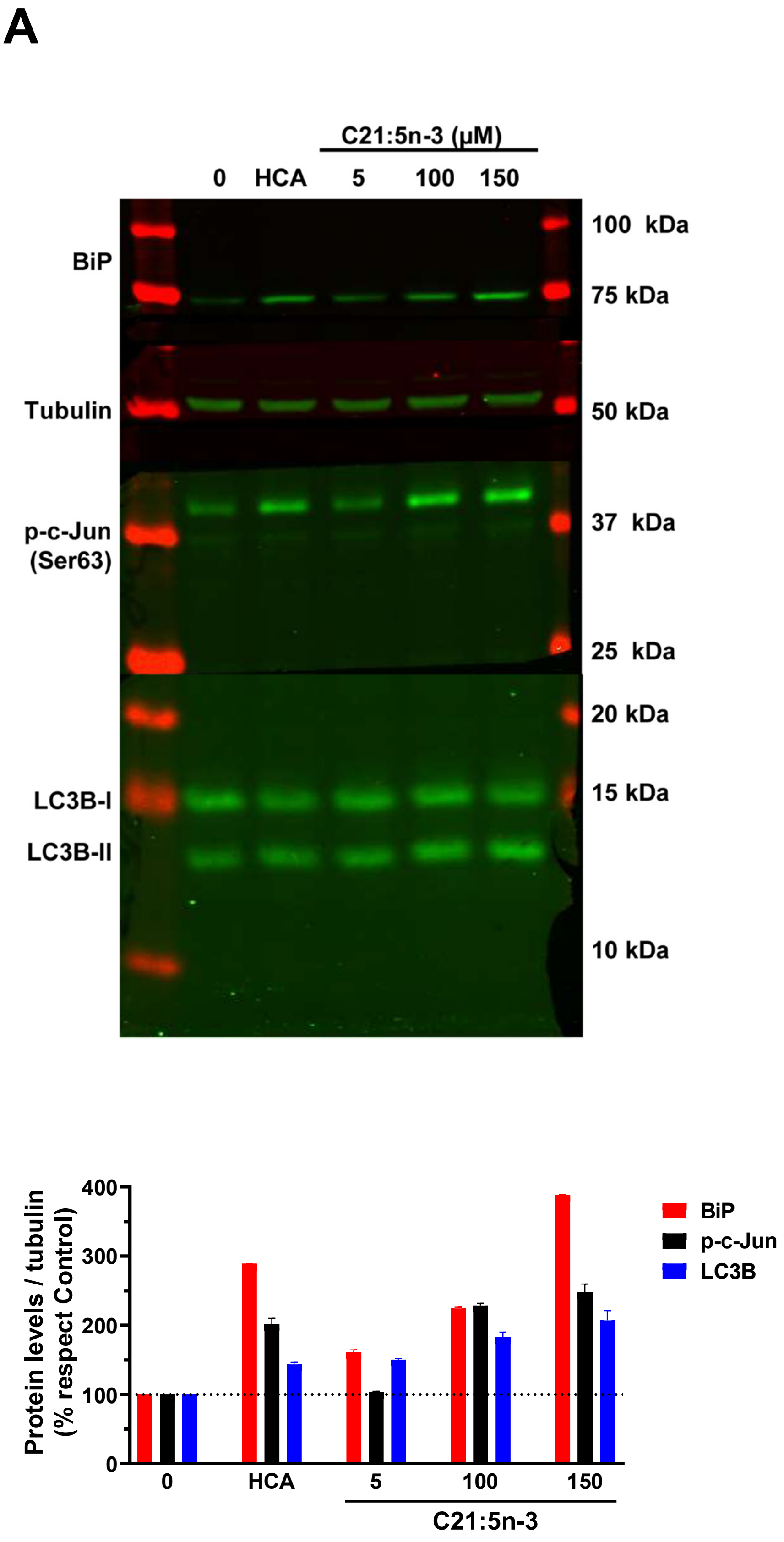
A
C21:5n-3 (μM)
0 HCA 5 100 150
BiP
Tubulin
p-c-Jun (Ser63)
LC3B-I
LC3B-II
100 kDa
75 kDa
50 kDa
37 kDa
25 kDa
20 kDa
15 kDa
10 kDa
Protein levels / tubulin (% respect Control)
400
300
200
100
0
0
HCA
5
100
150
C21:5n-3
BiP
p-c-Jun
LC3B

Figure 6.

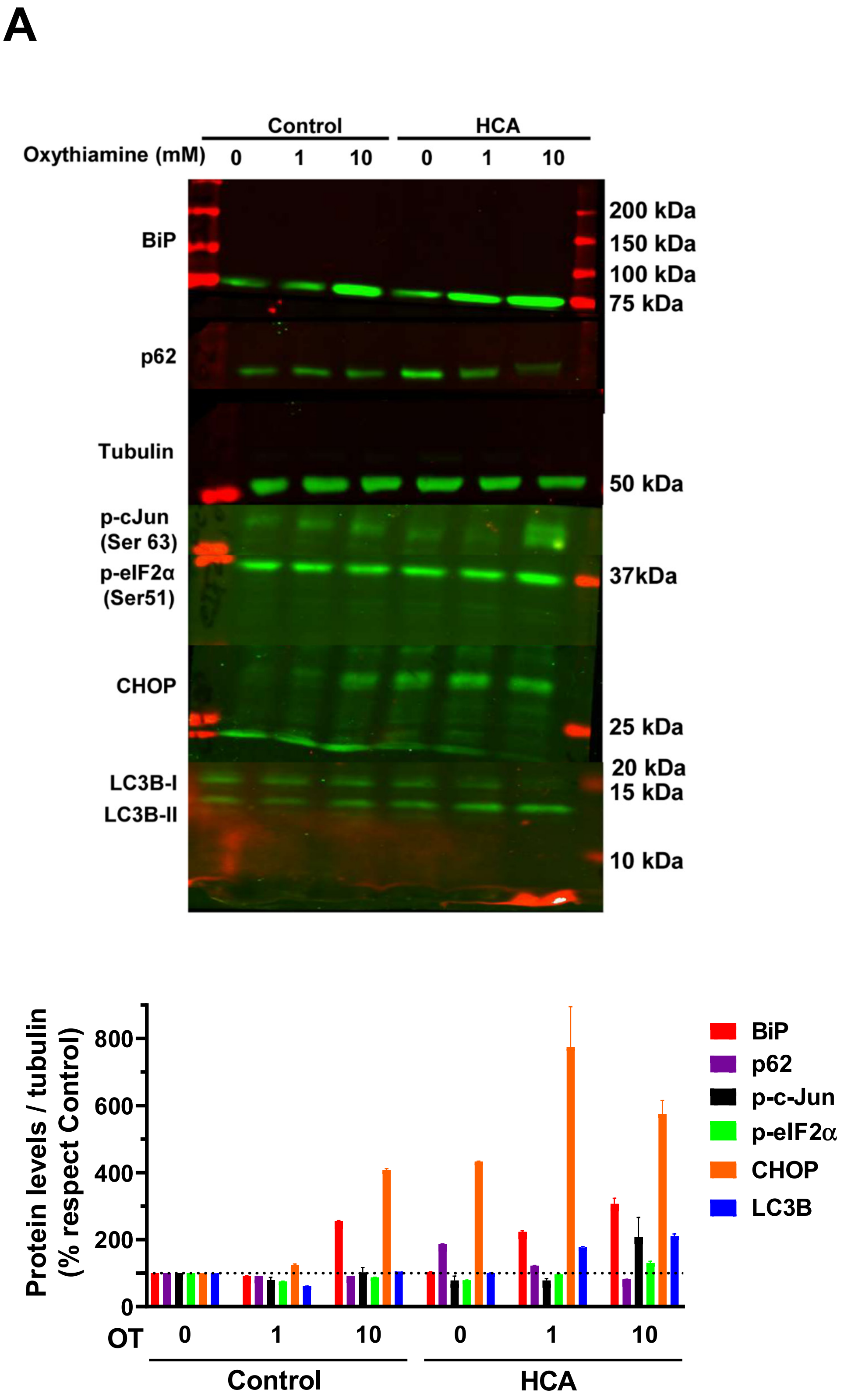
A
Control
HCA
Oxythiamine (mM) 0 1 10 0 1 10
BiP
p62
Tubulin
p-cJun (Ser 63)
p-eIF2α (Ser51)
CHOP
LC3B-I
LC3B-II
200 kDa
150 kDa
100 kDa
75 kDa
50 kDa
37kDa
25 kDa
20 kDa
15 kDa
10 kDa
Protein levels / tubulin (% respect Control)
800
600
400
200
0
OT 0 1 10 0 1 10
Control
HCA
BiP
p62
p-c-Jun
p-eIF2α
CHOP
LC3B

Figure 7.

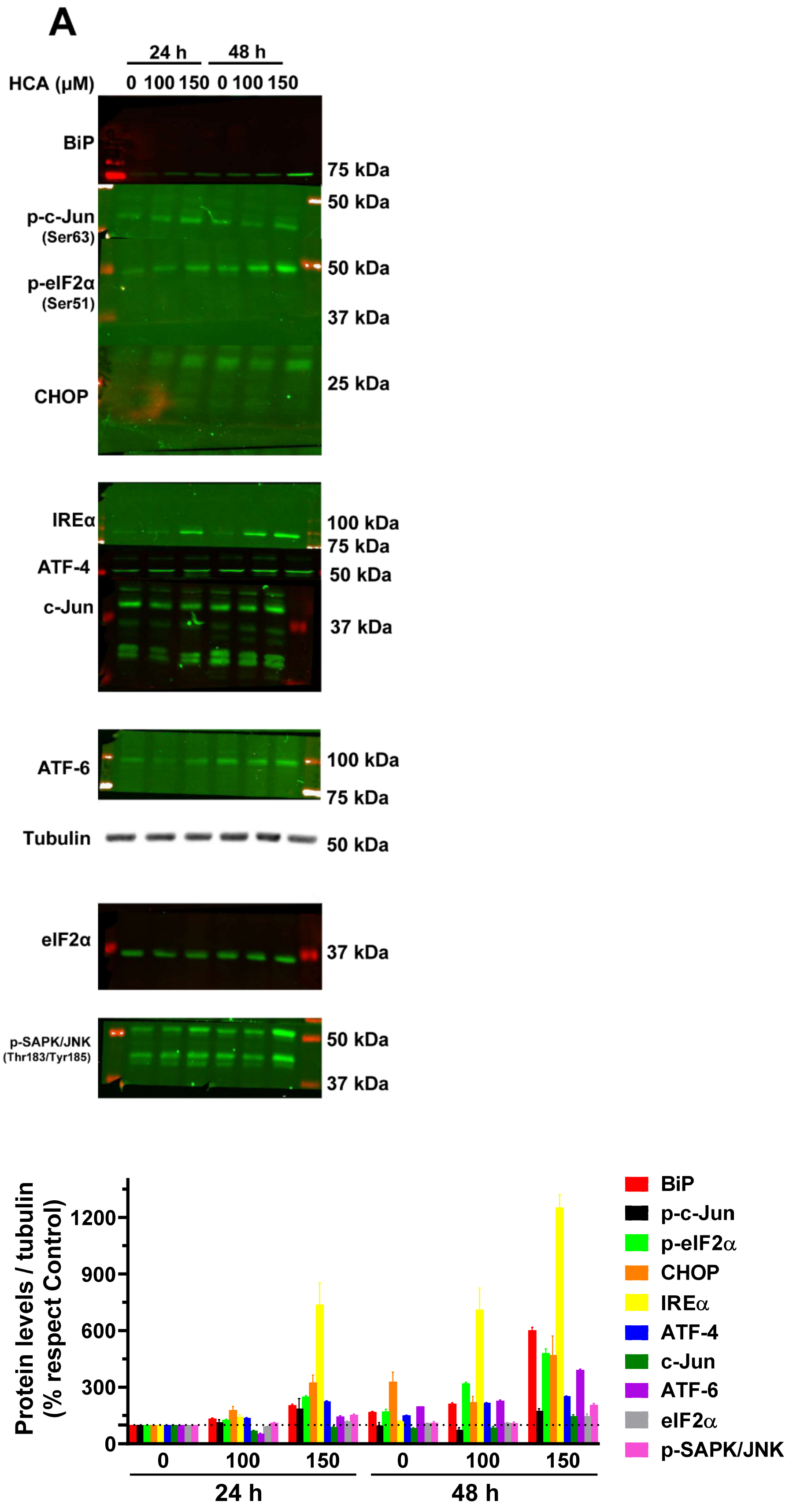

A
24 h
48 h
HCA (μM)
0 100 150 0 100 150
BiP
p-c-Jun (Ser63)
p-eIF2α (Ser51)
CHOP
IREα
ATF-4
c-Jun
ATF-6
Tubulin
eIF2α
p-SAPK/JNK (Thr183/Tyr185)
75 kDa
50 kDa
50 kDa
37 kDa
25 kDa
100 kDa
75 kDa
50 kDa
37 kDa
100 kDa
75 kDa
50 kDa
37 kDa
50 kDa
37 kDa
Protein levels / tubulin (% respect Control)
1200
900
600
300
0
0
100
150
0
100
150
24 h
48 h
BiP
p-c-Jun
p-eIF2α
CHOP
IREα
ATF-4
c-Jun
ATF-6
eIF2α
p-SAPK/JNK

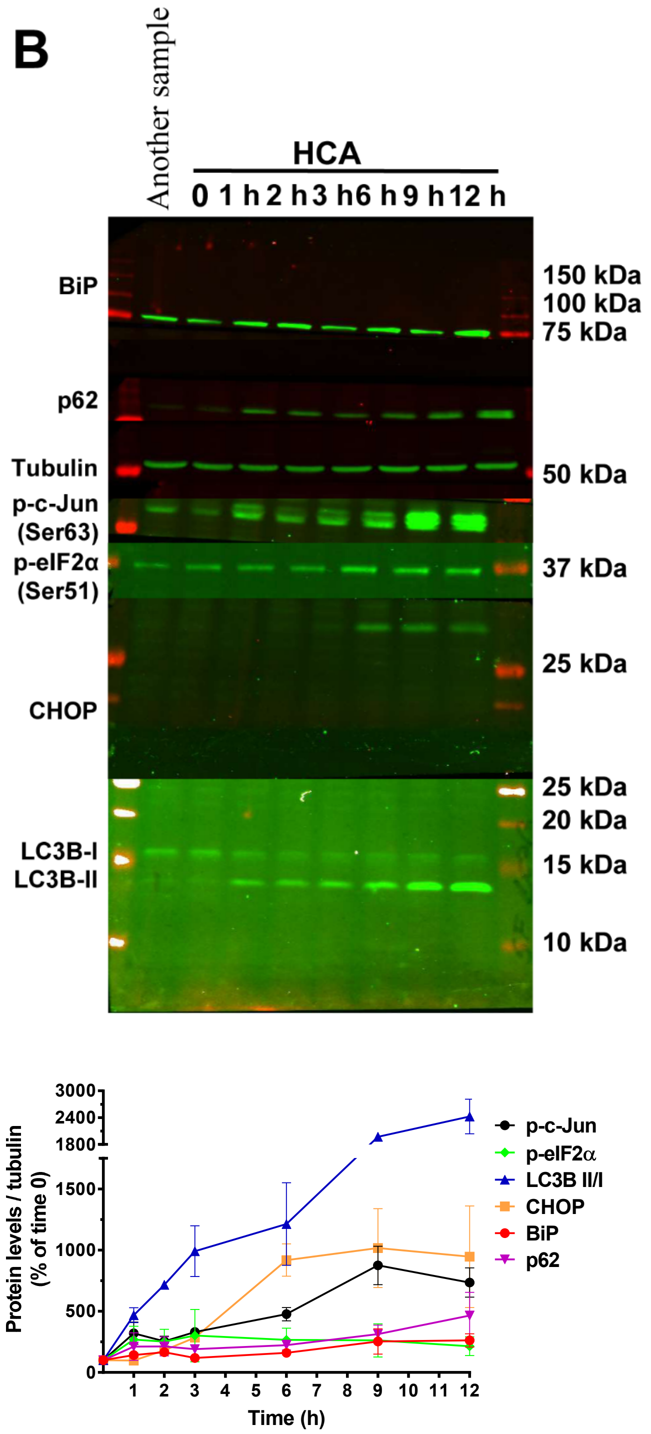
B
Another sample
HCA
0 1 h 2 h 3 h 6 h 9 h 12 h
BiP
p62
Tubulin
p-c-Jun (Ser63)
p-eIF2α (Ser51)
CHOP
LC3B-I
LC3B-II
150 kDa
100 kDa
75 kDa
50 kDa
37 kDa
25 kDa
25 kDa
20 kDa
15 kDa
10 kDa
Protein levels / tubulin (% of time 0)
Time (h)
p-c-Jun
p-eIF2α
LC3B II/I
CHOP
BiP
p62

Figure S2.

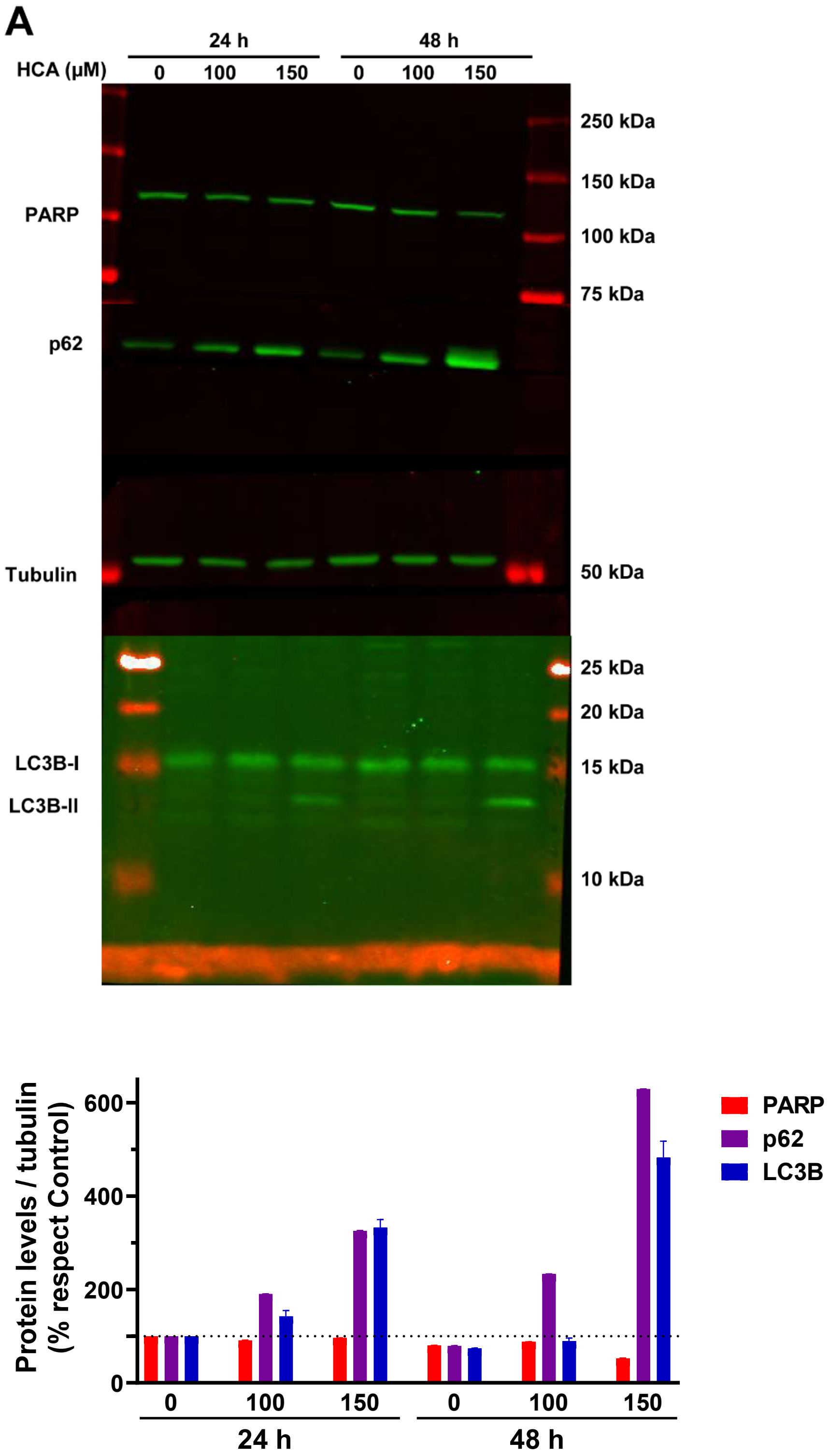

Figure S3.

Figure S5. Densitometric analysis of western blot results presented in Figure 2, Figure 3, Figure 4, Figure 6, Figure 7, Figure S2 and Figure S3. Western blots were normalized to α-tubulin and densitometric analysis was performed using image processing software. Values are mean ± standard error of the mean of independent experiments and values in the treated cells were considered relative to those of the untreated control cells. Student's t-test: *$p < 0.05$ with respect to the controls. BafA1: bafilomycin; HCA: 2-hydroxycervonic acid; OT: oxythiamine; SP600125: JNK (C-Jun N-Terminal Kinase 1) inhibitor; C21:5n-3: heneicosapentaenoic acid; ATF:

Activating Transcription Factor; BiP: a.k.a. GRP78, glucose-regulated protein 78; CHOP: a.k.a. DDIT3, DNA Damage Inducible Transcript 3; c-Jun: Jun Proto-Oncogene, AP-1 Transcription Factor Subunit; eIF2a: Eukaryotic Translation Initiation Factor 2A; IREα: inositol-requiring enzyme 1; LC3B: a.k.a. ATG8F, Microtubule Associated Protein 1 Light Chain 3 Beta; p62: a.k.a. SQSTM1, Sequestosome 1; PARP: Poly(ADP-Ribose) SAPK/JNK: Polymerase; Stress-activated protein kinases/Jun amino-terminal kinases; + : presence of the drug indicated in the figure; – : absence of the drug indicated in the figure.
